# PPIxGPN: plasma proteomic profiling of neurodegenerative biomarkers with protein–protein interaction-based eXplainable graph propagational network

**DOI:** 10.1093/bib/bbaf213

**Published:** 2025-05-29

**Authors:** Sunghong Park, Dong-gi Lee, Juhyeon Kim, Seung Ho Kim, Hyeon Jin Hwang, Hyunjung Shin, Hyun Goo Woo

**Affiliations:** Department of Physiology, Ajou University School of Medicine, Worldcup-ro 164, Yeongtong-gu, Suwon, 16499, Republic of Korea; Department of Biostatistics, Epidemiology and Informatics, Perelman School of Medicine, University of Pennsylvania, Philadelphia, PA 19104, USA; Department of Industrial Engineering, Ajou University, Worldcup-ro 206, Yeongtong-gu, Suwon, 16499, Republic of Korea; Department of Data-Centric Problem Solving Research, Korea Institute of Science and Technology Information, Daehak-ro 245, Yuseong-gu, Daejeon, 34141, Republic of Korea; Department of Physiology, Ajou University School of Medicine, Worldcup-ro 164, Yeongtong-gu, Suwon, 16499, Republic of Korea; Department of Biomedical Science, Graduate School of Ajou University, Worldcup-ro 164, Yeongtong-gu, Suwon, 16499, Republic of Korea; Department of Physiology, Ajou University School of Medicine, Worldcup-ro 164, Yeongtong-gu, Suwon, 16499, Republic of Korea; Department of Biomedical Science, Graduate School of Ajou University, Worldcup-ro 164, Yeongtong-gu, Suwon, 16499, Republic of Korea; Department of Industrial Engineering, Ajou University, Worldcup-ro 206, Yeongtong-gu, Suwon, 16499, Republic of Korea; Department of Artificial Intelligence, Ajou University, Worldcup-ro 206, Yeongtong-gu, Suwon, 16499, Republic of Korea; Department of Physiology, Ajou University School of Medicine, Worldcup-ro 164, Yeongtong-gu, Suwon, 16499, Republic of Korea; Department of Biomedical Science, Graduate School of Ajou University, Worldcup-ro 164, Yeongtong-gu, Suwon, 16499, Republic of Korea; Ajou Translational Omics Center, Research Institute for Innovative Medicine, Ajou University Medical Center, Worldcup-ro 164, Yeongtong-gu, Suwon, 16499, Republic of Korea

**Keywords:** neurodegenerative diseases, blood-based biomarkers, protein–protein interaction, graph neural network, explainable machine learning

## Abstract

Neurodegenerative diseases involve progressive neuronal dysfunction, requiring the identification of specific pathological features for accurate diagnosis. While cerebrospinal fluid analysis and neuroimaging are commonly used, their invasive nature and high costs limit clinical applicability. Recently advances in plasma proteomics offer a less invasive and cost-effective alternative, further enhanced by machine learning (ML). However, most ML-based studies overlook synergetic effects from protein–protein interactions (PPIs), which play a key role in disease mechanisms. Although graph convolutional network and its extensions can utilize PPIs, they rely on locality-based feature aggregation, overlooking essential components and emphasizing noisy interactions. Moreover, expanding those methods to cover broader PPIs results in complex model architectures that reduce explainability, which is crucial in medical ML models for clinical decision-making. To address these challenges, we propose Protein–Protein Interaction-based eXplainable Graph Propagational Network (PPIxGPN), a novel ML model designed for plasma proteomic profiling of neurodegenerative biomarkers. PPIxGPN captures synergetic effects between proteins by integrating PPIs with independent effects of proteins, leveraging globality-based feature aggregation to represent comprehensive PPI properties. This process is implemented using a single graph propagational layer, enabling PPIxGPN to be configured by shallow architecture, thereby PPIxGPN ensures high model explainability, enhancing clinical applicability by providing interpretable outputs. Experimental validation on the UK Biobank dataset demonstrated the superior performance of PPIxGPN in neurodegenerative risk prediction, outperforming comparison methods. Furthermore, the explainability of PPIxGPN facilitated detailed analyses of the discriminative significance of synergistic effects, the predictive importance of proteins, and the longitudinal changes in biomarker profiles, highlighting its clinical relevance.

## Introduction

Neurodegenerative diseases are characterized by the progressive dysfunction and loss of neurons in the brain and nervous system. Accurate diagnosis of these conditions depends on identifying specific pathological features [1]. For example, dementia, the most prevalent neurodegenerative disease, is categorized into several subtypes based on distinct neuropathological hallmarks. Alzheimer’s disease (AD) is associated with β-amyloid (Aβ) deposition and tau pathology, and Parkinson’s disease (PD) and Lewy body dementia are related to α-synuclein pathology [2]. Traditionally, the primary methods for identifying these neuropathological features have included cerebrospinal fluid (CSF) biomarkers and neuroimaging. CSF biomarkers, such as the Aβ_42_/Aβ_40_ ratio (Aβ_42/40_) and phosphorylated tau (pTau), are employed to evaluate nervous system conditions, diagnose neurological disorders, and monitor disease progression [3]. However, the invasive nature of CSF collection poses risks of severe complications. Neuroimaging provides objective evidence of Aβ deposition or tau tracer detection via positron emission tomography and assesses hippocampal or medial temporal neurodegeneration through magnetic resonance imaging [4]. Despite their utility, these methods are associated with high costs. As a result, in recent decades, substantial research efforts have been directed toward developing diagnostic biomarkers to better differentiate the pathophysiological features of neurodegenerative diseases [5].

Recently, the advent of plasma biomarkers has provided a less invasive and more cost-effective alternative for identifying diverse neuropathological features through the assessment of multiple biomarkers from a single sample [6]. There are four key plasma biomarkers associated with neurodegeneration: Aβ_42/40_, pTau, neurofilament light (NfL), and glial fibrillary acidic protein (GFAP). Aβ_42/40_ is widely used and strongly related to cerebral amyloid pathology and neuronal damage [7]. The blood levels of pTau significantly correlate with Aβ accumulation and clinical severity in AD [8]. NfL serves as a promising biomarker of neurodegeneration and neuroaxonal damage, reflecting active brain pathology [9]. GFAP is found to be associated with neuroinflammation and plays a key role in assessing various aspects of the astrocytic response in neurological disorders [10]. Accordingly, plasma proteomic profiling of these biomarkers offers a comprehensive understanding of neuropathological features, enabling precise diagnoses of neurodegenerative diseases.

Furthermore, the application of machine learning (ML) has significantly advanced plasma proteomic profiling of neurodegenerative biomarkers with more sophisticated analytical approaches [11]. However, many existing studies primarily focus on the independent effects of proteins, relying solely on expression data while often overlooking their interactions [12]. This oversight fails to capture the collective contributions of multiple proteins with small effect sizes, which can influence disease progression through their interactions [13]. Synergetic effects arising from protein–protein interactions (PPIs) play a crucial role in disease mechanisms, emphasizing the importance of PPI-based assessment of disease risk for understanding molecular pathogenesis [14]. Neurodegenerative diseases involve complex interactions between various molecular pathways, further underscoring the critical role of PPIs [15]. Additionally, advancements in PPI databases have broadened their coverage to nearly the entire proteome, facilitating the adoption of computational approaches based on PPI networks [16]. Consequently, the significance of ML-based plasma proteomic profiling of neurodegenerative biomarkers, enhanced by leveraging PPIs, is becoming increasingly evident.

One well-established ML approach for modeling the PPI network is graph neural network (GNN), particularly graph convolutional network (GCN) [17], where proteins are represented as nodes, PPIs as edges, and protein expression values as features. GCN leverages graph convolutional layers to aggregate feature information from one-hop neighboring nodes, enabling it to capture local properties of PPIs. However, it struggles to account for global properties of PPIs and multi-hop relationships between proteins [18]. To overcome this limitation, GCN has been adapted for multi-hop feature aggregation. Approaches like simple graph convolution (SGC) [19] utilize the higher-order graph convolutional filter to aggregate features from distant nodes. More advanced models, including Exponential Graph Convolution (EGC) and Linear Graph Convolution (LGC) [20], enhance SGC by incorporating more sophisticated graph Laplacian computations. Additionally, methods with parallelized graph convolutional architectures, such as MixHop [21] and its derivatives, Universal GCN (UGCN) [22] and Mixed-order GCN (MOGCN) [23], improve their ability to model a wider range of PPIs by incorporating multi-hop feature aggregation.

While the extended versions of GCN can account for a somewhat broader range of PPIs, they nevertheless fail to encompass the full range of properties present in the PPI network, where the locality-based feature representation of PPIs gives rise to a couple of issues. Firstly, the structural properties of the PPI network are overlooked. The PPI network is intricate and hierarchical, comprising various subnetworks. By focusing exclusively on interactions between nearby proteins, important features and modules within the entire PPI network may be overlooked [24, 25]. Secondly, the noisy interactions in the PPI network are emphasized. Since the PPI network incorporates data from diverse experimental sources, it is susceptible to some noisy interactions [26, 27]. The locality-based representation of PPIs may potentially exaggerate these noisy interactions. Accordingly, GCN-based models necessitate globality-based feature aggregation to address these issues and reflect the comprehensive properties of the PPI network.

Although the re-extension of GCN-based models can partially mitigate the aforementioned issues, it remains a provisional solution that fails to capture the comprehensive properties of the PPI network. Moreover, this approach necessitates a more complex model architecture, which compromises the explainability of the model and its outcomes [28]. Explainability is crucial for understanding the predictive processes of ML models, particularly in the medical field, where decisions directly impact on patient care [29]. Explainability improves the transparency of ML models, enabling healthcare professionals to trust and effectively integrate them into clinical decision-making [30]. Furthermore, it provides deeper insights into biological processes and disease mechanisms, aiding in understanding disease pathophysiology and advancing precision medicine [31, 32]. Therefore, to ensure high explainability, the globality-based feature aggregation, which comprehensively captures the properties of the PPI network, should be implemented using shallow model architecture.

**Figure 1:**
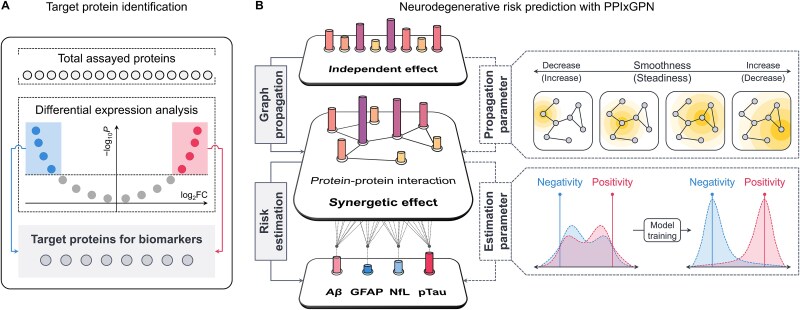
Overview of the proposed method. Our study framework comprises target protein identification and neurodegenerative risk prediction. (a) The proposed method first identifies DEPs associated with biomarkers, selecting those common across all biomarkers as target proteins. (b) PPIxGPN then integrates the independent effects of these proteins with the PPI network to predict individual risks for four biomarkers, consisting of graph propagation and risk estimation, each implemented by specific model parameters. The graph propagation parameter regulates the propagation intensity within the PPI network for each protein, while the risk estimation parameter fine-tunes protein effect sizes for each biomarker, ensuring a distinct separation between positive and negative diagnosis groups.

In this study, we propose a novel ML method, called *Protein–Protein Interaction-based eXplainable Graph Propagational Network* (PPIxGPN), for plasma proteomic profiling of neurodegenerative biomarkers, including Aβ, GFAP, NfL, and pTau. The proposed method leverages the PPI network by utilizing a GNN-based architecture, enabling it to capture synergetic effects between proteins and apply them to predict risks for the biomarkers. This process is implemented using a single graph propagational layer, a key component of the proposed method, allowing PPIxGPN to be configured by shallow architecture with only two layers, along with a fully connected layer for risk prediction. Accordingly, PPIxGPN reflects the comprehensive properties of the PPI network through globality-based feature aggregation while preserving an intuitive architecture that enhances the explainability of both the model and its outcomes. As illustrated in Fig. 1, our study framework consists of target protein identification and neurodegenerative risk prediction. The proposed method first identifies differentially expressed proteins (DEPs) associated with the biomarkers. Among these DEPs, those common to all biomarkers are identified as target proteins. Subsequently, PPIxGPN integrates the independent effects of target proteins with the PPI network to predict individual risks for four biomarkers. PPIxGPN comprises two processes: graph propagation and risk estimation, with model parameters defined for each. The parameter for graph propagation controls the propagation intensity within the PPI network for each protein, while the parameter for risk estimation optimizes the effect size of target proteins for each biomarker, ensuring a clear distinction between positive and negative diagnosis groups. The main contributions of the proposed method are summarized as follows:

- We introduce a novel method, called PPIxGPN, for plasma proteomic profiling of neurodegenerative biomarkers, which predict the individual risks for Aβ, GFAP, NfL, and pTau by identifying target proteins that are significantly expressed across all four biomarkers.
- PPIxGPN integrates the PPI network with the independent effects of target proteins for the biomarkers, effectively capturing synergetic effects among proteins, and by incorporating globality-based feature aggregation, PPIxGPN reflects the comprehensive properties of the PPI network.
- PPIxGPN is designed with shallow architecture including only two layers, ensuring high model explainability, thereby enhances clinical applicability by providing interpretable outputs that not only improve neurodegenerative risk prediction but also help understand the biological significance of PPIs and their contributions to disease progression.

## Protein-protein interaction-based eXplainable graph propagational network

### Overall process

Given that $d$ and $n$ are the numbers of target proteins and study participants, respectively, the data matrix for the independent effects, denoted by $\mathbf{X}\in{\mathbb{R}}^{d\times n}$, consists of the preprocessed expression data for the target proteins. PPIxGPN first extracts the synergetic effects of target proteins, denoted as $\mathbf{Z}\in{\mathbb{R}}^{d\times n}$, by propagating the independent effects onto the PPI network $\mathbf{W}\in{\mathbb{R}}^{d\times d}$. In this process, the propagation parameter $\boldsymbol{\phi} \in{\mathbb{R}}^d$ is applied to the proteins for individually controlling the intensity of propagation. Subsequently, the synergetic effect is applied to the estimation parameter, denoted as ${\boldsymbol{\Theta}}_{\ast}\in{\mathbb{R}}^d$ ($\ast$: Aβ, GFAP, NfL, pTau), and the individual risks for neurodegenerative biomarkers, denoted as ${\mathbf{P}}_{\ast}\in{\mathbb{R}}^n$, are derived. The proposed method encompasses two-layered model architecture, including two parameter sets, $\boldsymbol{\phi}$ and ${\boldsymbol{\Theta}}_{\ast}$, which are optimized by comparing the predicted risk ${\mathbf{P}}_{\ast}$ with the real diagnosis, denoted as ${\mathbf{Y}}_{\ast}\in{\mathbb{R}}^n$. Notations for PPIxGPN are summarized in Table 1.

**Table 1:** Summarized description for notation of PPIxGPN.

Notation	Description
$n$	Number of study participants
$d$	Number of target proteins
$\mathbf{X}\in{\mathbb{R}}^{d\times n}$	Independent effect of target proteins
$\mathbf{Z}\in{\mathbb{R}}^{d\times n}$	Synergetic effect of target proteins
$\mathbf{W}\in{\mathbb{R}}^{d\times d}$	PPI network for target proteins
$\boldsymbol{\phi} \in{\mathbb{R}}^d$	Propagation parameter set of PPIxGPN
${\boldsymbol{\Theta}}_{\ast}\in{\mathbb{R}}^d$	Estimation parameter set of PPIxGPN
${\mathbf{P}}_{\ast}\in{\mathbb{R}}^n$	Predicted risk set for biomarkers
${\mathbf{Y}}_{\ast}\in{\mathbb{R}}^n$	Real diagnosis label set for biomarkers
$\ast$	Aβ, GFAP, NfL, pTau

### Model implementation

PPIxGPN consists of two processes: *graph propagation* and *risk estimation*, where the former extracts the synergetic effect, while the latter derives the individual risks for neurodegenerative biomarkers. The graph propagation process transforms the independent effect into the synergetic effect that accounts for PPIs. Here, the “independent effect” refers to the expression value of each protein, capturing only its own expression level without considering PPIs. To incorporate PPIs, the proposed method represents the synergetic effect via the following objective function:


(1) \begin{equation*} \sum_{i\sim j}{\mathbf{W}}^{\left(i,j\right)}{\left({\mathbf{Z}}^{(i)}-{\mathbf{Z}}^{(j)}\right)}^2+\sum{\boldsymbol{\phi}}^{(i)}{\left({\mathbf{Z}}^{(i)}-{\mathbf{X}}^{(i)}\right)}^2. \end{equation*}

In Equation (1), the first term enhances “smoothness,” ensuring that adjacent proteins have similar representation values. The second term enhances “steadiness,” preventing the newly generated synergetic effect ($\mathbf{Z}$) from deviating excessively from the original independent effect ($\mathbf{X}$). The propagation parameter $\boldsymbol{\phi}$ is defined for each protein and can be viewed as a parameter that determines how much interaction information is exchanged with neighboring nodes—i.e. how extensively it propagates. The objective function in Equation (1) can be expressed in matrix form as shown below:


(2) \begin{equation*} \underset{\mathbf{Z}}{\min }\ {\mathbf{Z}}^{\mathrm{T}}\mathbf{LZ}+{\left(\mathbf{Z}-\mathbf{X}\right)}^{\mathrm{T}}\boldsymbol{\Phi} \left(\mathbf{Z}-\mathbf{X}\right) \end{equation*}

where $\boldsymbol{\Phi}$ is the diagonal matrix for $\boldsymbol{\phi}$, and $\mathbf{L}$ is the normalized graph Laplacian for $\mathbf{W}$, which is defined as $\mathbf{L}={\mathbf{I}}_d-{\mathbf{D}}^{-1/2}\mathbf{W}{\mathbf{D}}^{-1/2}$ with the diagonal matrix $\mathbf{D}=\mathrm{Diag}\left({\mathbf{D}}^{(i)}\right)$ from ${\mathbf{D}}^{(i)}={\sum}_k{\mathbf{W}}^{\left(i,k\right)}$ and the identity matrix ${\mathbf{I}}_d\in{\mathbb{R}}^{d\times d}$. The solution of Equation (2) is obtained in the closed form as below:


(3) \begin{equation*} \mathbf{Z}={\left(\boldsymbol{\Phi} +\mathbf{L}\right)}^{-1}\boldsymbol{\Phi} \mathbf{X} \end{equation*}

As a result, the graph propagation process simultaneously propagates protein expression (the independent effect) across the entire PPI network, producing a new representation (the synergetic effect) that accounts for relationships with adjacent nodes. Because this global (all-hop) propagation is computed in closed form within a single layer, it can capture the complete properties of the PPI network in one pass—without stacking multiple layers.

Subsequently, in the risk estimation process, $\mathbf{Z}$ is combined with the estimation parameter ${\boldsymbol{\Theta}}_{\ast }$, and then, the individual risks for neurodegenerative biomarkers are derived by applying the logistic function as follows:


(4) \begin{equation*} {\mathbf{P}}_{\ast }=1/\left(1+{e}^{-{{\boldsymbol{\Theta}}_{\ast}}^{\mathrm{T}}\mathbf{Z}}\right) \end{equation*}

### Parameter optimization

PPIxGPN includes two parameter sets: $\boldsymbol{\phi}$ for the graph propagation and ${\boldsymbol{\Theta}}_{\ast }$ for the risk estimation. These parameters are trained to minimize the binary cross-entropy loss ${\mathcal{L}}_{\ast }$ as represented below:


(5) \begin{equation*} {\mathcal{L}}_{\ast }=\frac{1}{n}\left\{\left({{\mathbf{Y}}_{\ast}}^{\mathrm{T}}\log{\mathbf{P}}_{\ast}\right)+{\left({\mathbf{1}}_n-{\mathbf{Y}}_{\ast}\right)}^{\mathrm{T}}\log \left({\mathbf{1}}_n-{\mathbf{P}}_{\ast}\right)\right\} \end{equation*}

Accordingly, the objective function for PPIxGPN is defined as follows:


(6) \begin{equation*} \underset{\boldsymbol{\phi}, \kern0.5em {\boldsymbol{\Theta}}_{\ast }}{\mathrm{argmin}}\kern0.5em \sum{\mathcal{L}}_{\ast }+\delta \mathcal{R} \end{equation*}

where $\mathcal{R}={\left\Vert \boldsymbol{\phi} \right\Vert}_2^2+\sum{\left\Vert{\boldsymbol{\Theta}}_{\ast}\right\Vert}_2^2$ stands for regularizing the complexity of parameters, and $\delta$ is the hyper-parameter for $\mathcal{R}$. The objective function in Equation (6) is optimized by using the gradient descent method [33].

**Minimization over**  ${\boldsymbol{\Theta}}_{\ast }$: to find gradient *w.r.t.* the estimation parameter ${\boldsymbol{\Theta}}_{\ast }$, the derivatives of two terms in Equation (6) *w.r.t.*  ${\boldsymbol{\Theta}}_{\ast }$ are firstly obtained as below:


$$ \frac{\partial{\mathcal{L}}_{\ast }}{\partial{\boldsymbol{\Theta}}_{\ast }}=\frac{1}{n}\mathbf{Z}{\left({\mathbf{P}}_{\ast }-{\mathbf{Y}}_{\ast}\right)}^{\mathrm{T}},\kern0.5em \frac{\partial \mathcal{R}}{\partial{\boldsymbol{\Theta}}_{\ast }}=2\delta{\boldsymbol{\Theta}}_{\ast } $$

Then, the gradient *w.r.t.*  ${\boldsymbol{\Theta}}_{\ast }$ is derived as follows:


(7) \begin{equation*} \nabla{\boldsymbol{\Theta}}_{\ast }=\frac{1}{n}\mathbf{Z}{\left({\mathbf{P}}_{\ast }-{\mathbf{Y}}_{\ast}\right)}^{\mathrm{T}}+2\delta{\boldsymbol{\Theta}}_{\ast } \end{equation*}

**Minimization over**  $\boldsymbol{\phi}$: to find the gradient *w.r.t.*, the propagation parameter $\boldsymbol{\phi}$, the derivative of $\mathcal{L}$  *w.r.t.*  $\mathbf{Z}$ is firstly obtained as follows:


(8) \begin{equation*} \frac{\partial \mathcal{L}}{\partial \mathbf{Z}}=\sum \frac{\partial{\mathcal{L}}_{\ast }}{\partial \mathbf{Z}}=\frac{1}{n}\sum{\boldsymbol{\Theta}}_{\ast}\left({\mathbf{P}}_{\ast }-{\mathbf{Y}}_{\ast}\right) \end{equation*}

Next, the derivative of $\mathbf{Z}$  *w.r.t.*  $\boldsymbol{\Phi}$ is obtained as follows:


(9) \begin{equation*} \frac{\partial \mathbf{Z}}{\partial \boldsymbol{\Phi}}={\mathbf{X}}^{\mathrm{T}}{\left(\boldsymbol{\Phi} +\mathbf{L}\right)}^{-1}\left\{{\mathbf{I}}_p-{\left(\boldsymbol{\Phi} +\mathbf{L}\right)}^{-1}\boldsymbol{\Phi} \right\} \end{equation*}

By combining Equation (8) with Equation (9), $\partial \mathcal{L}/\partial \boldsymbol{\Phi}$ is indicated as below:


(10) \begin{equation*} \frac{\partial \mathcal{L}}{\partial \boldsymbol{\Phi}}=\frac{1}{n}\left\{\sum{\boldsymbol{\Theta}}_{\ast}\left({\mathbf{P}}_{\ast }-{\mathbf{Y}}_{\ast}\right)\right\}{\mathbf{X}}^{\mathrm{T}}{\left(\boldsymbol{\Phi} +\mathbf{L}\right)}^{-1}\left\{{\mathbf{I}}_p-{\left(\boldsymbol{\Phi} +\mathbf{L}\right)}^{-1}\boldsymbol{\Phi} \right\} \end{equation*}

Subsequently, the expression of $\boldsymbol{\Phi}$ is transformed to $\boldsymbol{\Phi} =\mathrm{Diag}\left(\boldsymbol{\phi} \right)=\boldsymbol{\phi} {\mathbf{1}}_d\odot{\mathbf{I}}_d$ by using an $d$-dimensional row vector ${\mathbf{1}}_d$, and then, the derivative of $\boldsymbol{\Phi}$  *w.r.t.*  $\boldsymbol{\phi}$ is indicated as below:


(11) \begin{equation*} \frac{\partial \boldsymbol{\Phi}}{\partial \boldsymbol{\phi}}={\mathbf{I}}_d{{\mathbf{1}}_d}^{\mathrm{T}} \end{equation*}

By combining Equation (10) with Equation (11), the derivative of $\mathcal{L}$  *w.r.t.*  $\boldsymbol{\phi}$ can be obtained as


$$\frac{\partial \mathcal{L}}{\partial \boldsymbol{\phi}}=\frac{1}{n}\left\{\sum{\boldsymbol{\Theta}}_{\ast}\left({\mathbf{P}}_{\ast }-{\mathbf{Y}}_{\ast}\right)\right\}{\mathbf{X}}^{\mathrm{T}}{\left(\boldsymbol{\Phi} +\mathbf{L}\right)}^{-1}\left\{{\mathbf{I}}_d-{\left(\boldsymbol{\Phi} +\mathbf{L}\right)}^{-1}\boldsymbol{\Phi} \right\}\odot{\mathbf{I}}_d{{\mathbf{1}}_d}^{\mathrm{T}},$$

and finally, the gradient *w.r.t.*  $\boldsymbol{\phi}$ is derived as follows:


(12) \begin{eqnarray*} && \nabla \boldsymbol{\phi} =\frac{1}{n}\left\{\sum{\boldsymbol{\Theta}}_{\ast}\left({\mathbf{P}}_{\ast }-{\mathbf{Y}}_{\ast}\right)\right\}{\mathbf{X}}^{\mathrm{T}}{\left(\boldsymbol{\Phi} +\mathbf{L}\right)}^{-1}\left\{{\mathbf{I}}_d-{\left(\boldsymbol{\Phi} +\mathbf{L}\right)}^{-1}\boldsymbol{\Phi} \right\} \nonumber\\&& \quad\qquad \odot{\mathbf{I}}_d{{\mathbf{1}}_d}^{\mathrm{T}}+2\delta \boldsymbol{\phi} \end{eqnarray*}

The overall procedure for PPIxGPN is summarized in Algorithm 1.

**Algorithm 1:** PPIxGPN.

**Input:** Independent effects of target proteins $\mathbf{X}\in{\mathbb{R}}^{d\times n}$ PPI network for target proteins $\mathbf{W}\in{\mathbb{R}}^{d\times d}$ Real diagnosis for biomarkers ${\mathbf{Y}}_{\ast}\in{\mathbb{R}}^n$ ($\ast$: Aβ, GFAP, NfL, pTau) **Output:** Individual risks for biomarkers ${\mathbf{P}}_{\ast}\in{\mathbb{R}}^n$ Initialize parameters: $\boldsymbol{\phi} \in{\mathbb{R}}^d$ and ${\boldsymbol{\Theta}}_{\ast}\in{\mathbb{R}}^d$ ** While** (stopping criterion is not satisfied) * Feedforward* Graph propagation: synergetic effect $\mathbf{Z}$ by (3) Risk estimation: individual risks ${\mathbf{P}}_{\ast }$ by (4) * Loss functions* Binary cross-entropy loss ${\mathcal{L}}_{\ast }$ by (5) Regularization loss $\mathcal{R}$ by (6) *Backpropagation* Propagation parameter $\boldsymbol{\phi} :=\boldsymbol{\phi} -\eta \nabla \boldsymbol{\phi}$ by (7) Estimation parameter ${\boldsymbol{\Theta}}_{\ast }:={\boldsymbol{\Theta}}_{\ast }-\eta \nabla{\boldsymbol{\Theta}}_{\ast }$ by (12) ** End while** **Return** Individual risks for biomarkers ${\mathbf{P}}_{\ast }$

## Results

### Data description

#### Study participants

The dataset used in this study was sourced from the UK Biobank (UKB), which recruited over 500,000 participants aged 39–70 between 2006 and 2010, with long-term monitoring of their health outcomes (additional details can be at: https://biobank.ndph.ox.ac.uk/showcase/). From the UKB participants, 906 individuals with complete data on four neurodegenerative biomarkers (Aβ_42/40_, GFAP, NfL, and pTau_181_) were selected for the analytical cohort. Given that higher levels of GFAP, NfL, and pTau indicate increased risk, while lower levels of Aβ_42/40_ signify greater risk, the reciprocal of Aβ_42/40_ (Aβ_40/42_) was used in this study to maintain consistency in predictions. For each participant, the positivity and negativity for the biomarkers were determined using the “cutoff” [34] implemented in R (https://github.com/choisy/cutoff). Of the participants, 783 had follow-up data on the biomarkers, with an average follow-up duration of 3.25 years, enabling the assessment of longitudinal changes. The demographic characteristics of the study participants are presented in Table 2.

**Table 2:** Demographic characteristics of the study participants.

Characteristics	Total participants (*N* = 906)	Followed-up participants (*N* = 783)		
		Baseline	Follow-up	*P*-value^a^
Female, No. (%)	491 (54.2)	422 (53.9)		–
Education, med. (IQR)	7 (3–17)	7 (3–17)		–
Age, med. (IQR), year	58 (54–64)	58 (53–64)	61 (57–67)	<.0001
Aβ-positivity, No. (%)	192 (21.2)	162 (20.7)	265 (33.8)	<.0001
GFAP-positivity, No. (%)	175 (19.3)	155 (19.8)	205 (26.2)	.0027
NfL-positivity, No. (%)	227 (25.1)	189 (24.1)	236 (30.1)	.0075
pTau-positivity, No. (%)	212 (23.4)	182 (23.2)	163 (20.8)	.2470

^a^ *P*-values were calculated by comparing the baseline and follow-up measurements for each characteristic among the followed-up participants.

#### Plasma samples

The plasma samples of participants were profiled by the Olink Platform [35], where 1463 proteins were totally assayed (further details can be found at: https://olink.com/). For the initial data for protein expression, proteins with a missing frequency >5% were excluded, and missing values were estimated using the *k*-nearest neighbor method. Subsequently, protein expression levels were standardized through *Z*-score normalization and then scaled using the logistic function.

### Proteomic assays

#### Differentially expressed proteins

**Figure 2:**
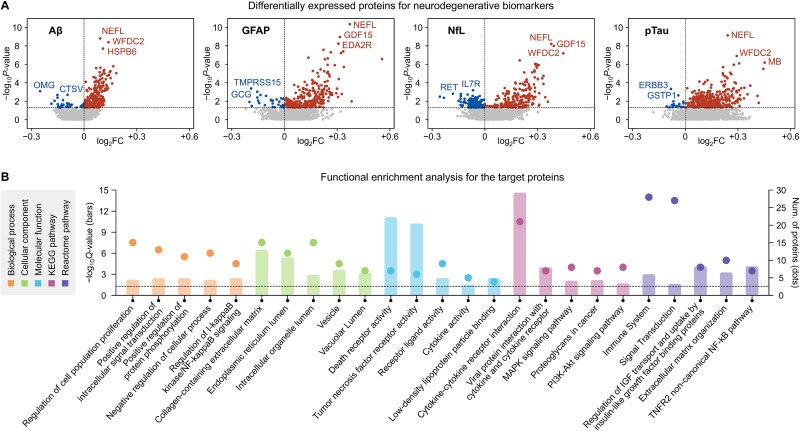
Proteomic assays of target proteins for neurodegenerative biomarkers. (a) Shows the differentially expressed proteins for neurodegenerative biomarkers. (b) Presents the results for the functional enrichment analysis for 113 proteins that were found to be commonly significant to all biomarkers.

Differential expression analysis (DEA) was conducted to identify proteins associated with neurodegenerative biomarkers among the total assayed proteins. The expression levels of each protein were compared between the case and control groups for the biomarkers, and the statistical significance of those differences is subsequently analyzed. To carry out this analysis, the “limma” R/Bioconductor package [36] was utilized. Of the 1412 proteins that passed quality control, DEA identified 237, 344, 411, and 440 DEPs for Aβ, GFAP, NfL, and pTau, respectively, using a *P*-value threshold of .05 (Fig. 2a). A total of 670 DEPs were associated with neurodegenerative biomarkers, with 113 proteins shared across all biomarkers. These shared DEPs were selected as the target proteins for this study. Among the target proteins, 104 were consistently upregulated across all biomarkers, with an average –log_10_ *P*-value of 3.14. NEFL showed the highest –log_10_ *P*-value (9.94), followed by WFDC2 (7.63) and GDF15 (6.89). Clinical studies have established significant associations between the plasma levels of these proteins and the pathogenesis of neurodegenerative diseases, including AD and PD [11, 37, 38]. Detailed results for the target proteins are provided in Supplementary Table S1.

**Figure 3:**
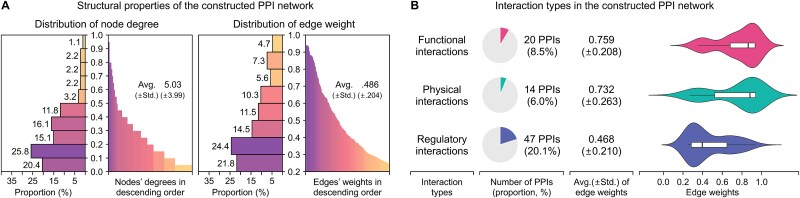
Structural properties and interaction types in the constructed PPI network. (a) Shows the structural properties of the PPI network for the 113 target proteins and (b) presents functional, physical, and regulatory interactions in the constructed PPI network.

#### Functional enrichment analysis

Subsequently, the functional enrichment analysis was performed on the target proteins for neurodegenerative biomarkers by employing the “Enrichr” [39] (https://maayanlab.cloud/Enrichr/). This analysis identified significant terms for the target proteins across five categories of functional annotations: three gene ontology (GO) domains (biological process, cellular component, and molecular function) and two pathway databases (KEGG [40] and Reactome [41]), with a thresholding *Q*-value of .05. Figure 2b shows the top five terms with high protein frequencies for each category. The findings revealed that the proteins were significantly associated with cytokines, the immune system, and signal transduction, highlighting the critical role of cytokines in influencing the central nervous system through various mechanisms, as well as the neuroinflammation they mediate in the development of neurodegenerative diseases [42, 43].

#### Protein–protein interaction network construction

The PPI network for the 113 target proteins was constructed based on the combined scores of PPIs obtained from the STRING database [44] (version 12.0, https://string-db.org/). In the constructed network, PPIs with a combined score >0.4 were included, and the edge weights between proteins were first standardized through *Z*-score normalization of the combined score, then scaled to values between 0 and 1 using a logistic function. As a result, the constructed PPI network included 234 interactions among 93 of the 113 proteins, each of which was connected by at least one edge, representing a network density of 5.47% (Supplementary Fig. S1). As shown in Fig. 3a, each protein was connected to an average of 5.03 other proteins, with TNFRSF1A having the highest node degree of 19, and the average weight of the 234 edges included in the network was 0.486. Furthermore, we identified PPIs corresponding to functional, physical, and regulatory interactions among the entire set of PPIs (Fig. 3b). Specifically, 20 PPIs were verified as functional interactions through the Reactome database (version 91) [41], 14 were identified as physical interactions using IntAct (release 249) [45] and BioGRID (release 4.4.240) [46] databases, and 47 were confirmed as regulatory interactions based on co-expression evidence in the STRING database.

**Figure 4:**
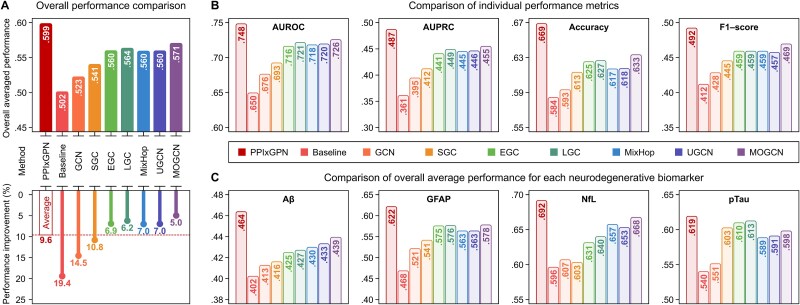
Performance comparison for predicting neurodegenerative risks. The performance of PPIxGPN was evaluated against the baseline model and seven GNN-based methods using AUROC, AUPRC, accuracy, and F1-score. (a) Compares the overall performance metric—Calculated as the average of AUROC, AUPRC, accuracy, and F1-score—and highlights the performance improvements achieved by PPIxGPN. (b) Presents the individual comparisons for each performance metric, and (c) shows the averages of overall performance metrics for each neurodegenerative biomarker.

### Performance evaluation

#### Experimental settings

We conducted three types of experiments to evaluate the performance of the proposed method. First, we compared its performance with that of the baseline model, which trained the independent effect, and seven GNN-based models—GCN [17], SGC [19], EGC [20], LGC [20], MixHop [21], UGCN [22], and MOGCN [23]—applicable to the PPI network. The maximum order of PPIs reflected in those models were 2 for GCN, 3 for SGC, 4 for EGC and MixHop, 5 for LGC and UGCN, and 6 for MOGCN, where a model with a maximum PPI order of *K* considers interactions up to the *K*-hop neighboring proteins in the PPI network. All models were trained using the ADAM optimizer [47] with a learning rate of 0.001. The performance was measured by the area under the receiving operating characteristic curve (AUROC), area under precision-recall curve (AUPRC), accuracy, and F1-score, with 100 iterations of five-fold cross-validation.

Second, we validated the performance improvement obtained by incorporating the synergetic effects derived from PPIxGPN into various ML models that had previously been used to learn the independent effects of proteins. In the blood protein-based diagnostic models, Logistic Regression Classifier, Linear SVM, Kernel SVM, Random Forest Classifier, AdaBoost, and XGBoost were each applied in [48–53], respectively. In addition to these five models, we employed seven more ML models—linear discriminant analysis, Quadratic Discriminant Analysis, Polynomial Support Vector Machines, *K*-Nearest Neighbors, Naïve Bayes Classifier, Decision Tree Classifier, Generalized Linear Model—in our experiments.

Last, we conducted an empirical analysis of the PPI network focusing on edge density and interaction types. At first, we examined how varying the edge density of the PPI network affects performance in predicting neurodegenerative risk. Specifically, from the constructed network, we randomly selected edges corresponding to 20%, 40%, 60%, and 80% of all PPIs to build respective PPI networks and applied our proposed method. For each proportion, we constructed 100 random networks and performed repeated experiments. The results were compared both to the outcome of learning only the independent effect and to the outcome of our proposed method when applying all PPIs in the constructed network. Next, we highlighted functional, physical, and regulatory interactions in the existing PPI network and constructed new networks accordingly, then applied PPIxGPN to investigate any changes in performance for neurodegenerative risk prediction. For each interaction type, we set the weights of the corresponding edges to 1, constructing three new PPI networks in total. We then applied our proposed method to these networks in the same manner as before.

#### Comparison results

The performance comparison results are illustrated in Fig. 4. By comparing an overall performance metric that averages AUROC, AUPRC, accuracy, and F1-score, PPIxGPN achieved the highest score of 0.599 (Fig. 4a), while that of the comparison methods ranged from 0.502 to 0.571, with PPIxGPN outperforming them by 9.6% on average. Among the comparison methods, MOGCN, with a maximum PPI order of 6, demonstrated the highest average performance of 0.571, while GCN, with a maximum PPI order of 2, exhibited the lowest average performance of 0.523. These findings suggest a positive correlation between the maximum PPI order and performance, with the proposed method achieving optimal results by incorporating global properties of the PPI network. Furthermore, an analysis of individual performance metrics revealed that the proposed method consistently outperformed the comparison methods, achieving superior results across all metrics (Fig. 4b). It was also observed that higher maximum PPI orders contributed to improved performance in individual metrics.

**Figure 5:**
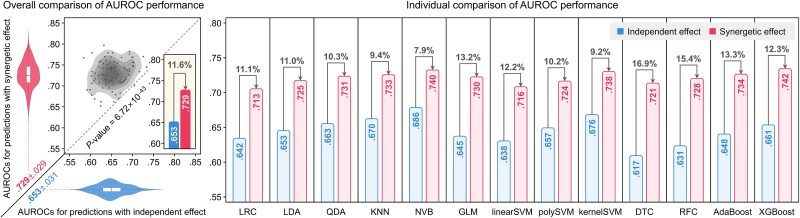
Prediction enhancement by incorporating synergetic effects. For 13 machine learning algorithms, the performance of neurodegenerative risk prediction based on learning the independent effect was compared with that based on learning the synergistic effect.

In addition, Fig. 4c shows the performance comparison for each neurodegenerative biomarker. The averages of overall performance metrics across all methods for Aβ, GFAP, NfL, and pTau were 0.428, 0.557, 0.639, and 0.590, respectively. The proposed method achieved the most optimal results for each biomarker, surpassing the comparison methods by an average of 9.7%, 13.8%, 9.6%, and 5.7% for Aβ, GFAP, NfL, and pTau, respectively. Among the biomarkers, NfL exhibited the highest performance, while Aβ demonstrated the lowest performance, with an average of 49.1% lower than the others. These results align with the understanding that plasma Aβ biomarkers have a lower signal-to-noise ratio and weaker associations with diagnostic outcomes, where clinical studies have highlighted the inherent complexity and variability of Aβ, influenced by subtle level variations and numerous confounding factors, contributing to diagnostic challenges in dementia [54–56]. Detailed results of the performance comparison are provided in Supplementary Table S2.

#### Prediction enhancements

Figure 5 presents a comparison of neurodegenerative risk prediction performance, based on training the independent and synergistic effects, across 13 different ML models. Models trained on the synergetic effect achieved an average AUROC of 0.729, representing an 11.6% improvement over the 0.653 average AUROC obtained from the independent effect. This difference was statistically significant, with a *P*-value of 6.72 × 10^−43^. These results demonstrate that the synergetic effect captured by PPIxGPN offers greater discriminative power in biomarker identification compared to the independent effect. Furthermore, our approach performs effectively even with relatively simple ML algorithms, suggesting its generalizability for broader applications.

**Figure 6:**
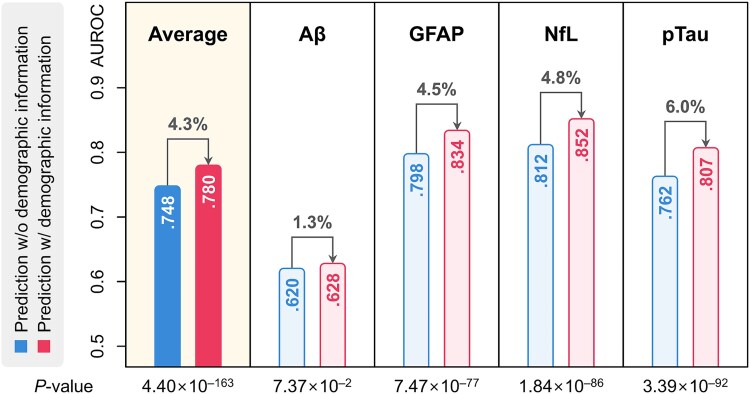
Prediction enhancement by integrating demographic information. The enhanced predictions were achieved by integrating demographic information, such as participants' age, sex, and education scores, into PPIxGPN.

In addition, prediction outcomes were further enhanced by incorporating demographic information, including participants' age, sex, and education scores, into PPIxGPN. As shown in Fig. 6, the enhanced PPIxGPN achieved an AUROC performance of 0.780, a 4.3% improvement over the original result, which was statistically significant with a *P*-value of 4.40 × 10^−163^. The most notable improvement was observed in pTau prediction, with a 6.0% increase, followed by a 4.8% improvement in NfL prediction. Apart from Aβ prediction, which showed the smallest performance gain, the AUROC improvements for the other three biomarkers were statistically significant. These findings indicate that PPIxGPN not only effectively integrates protein expression levels but also aligns well with demographic information, enhancing predictive performance by providing additional context. This capability improves the model's ability to identify patterns associated with neurodegenerative biomarkers and underscores its potential for robust neurodegenerative risk prediction.

**Figure 7:**
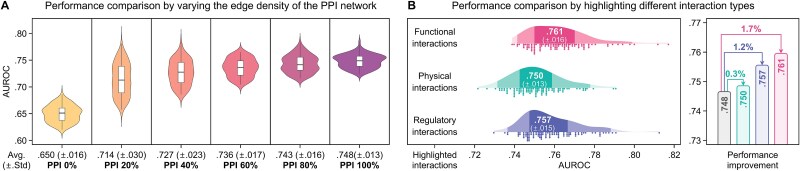
Empirical analysis of the PPI network. In predicting neurodegenerative risk: (a) shows the variation in the performance of PPIxGPN according to the edge density of the PPI network, while (b) compares the performance of PPIxGPN when functional, physical, and regulatory interactions are highlighted in the original network.

#### Empirical analysis

Figure 7 presents the results of our empirical analysis of the PPI network, focusing on edge density and interaction types. First, compared to neurodegenerative risk prediction based on learning only independent effects, incorporating synergetic effects between proteins via PPIs resulted in an average 12.9% increase in AUROC, with performance improvements ranging from 9.8% to 15.1% depending on edge density (Fig. 7a). The analysis also showed that higher edge density led to better predictive performance, where using all edges yielded the highest performance with an AUROC of 0.748, which is 4.8% higher than the AUROC of 0.714 achieved when only 20% of the edges were used. Next, when highlighting the edges corresponding to functional, physical, and regulatory interactions in the constructed PPI network, all three networks outperformed the original network, achieving an average 1.1% improvement in AUROC (Fig. 7b). Among these three interaction types, highlighting functional interactions produced the best result with an AUROC of 0.761, which was on average 1.7% higher than the original performance. These findings demonstrate that a model comprehensively accounting for PPIs can enhance the accuracy of neurodegenerative disease risk prediction, suggesting that adopting differentiated strategies based on interaction types may be effective.

**Figure 8:**
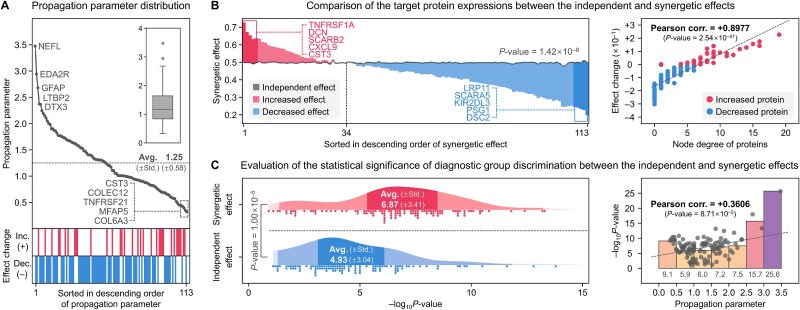
Discriminative significance of the synergetic effect. (a) Illustrates the distribution of propagation parameters for the target proteins. (b) Compares the protein expressions between the independent and synergetic effects. (c) Evaluates the statistical significance of diagnostic group discrimination between the independent and synergetic effects.

### Explanatory analyses

#### Discriminative significance of the synergetic effect

We evaluated the discriminative significance of the synergetic effect derived from PPIxGPN. Initially, the propagation parameters of the target proteins averaged 1.25, with NEFL showing the highest value at 3.47 (Fig. 8a and Supplementary Table S3). A correlation of −0.1849 (*P*-value = 4.99 × 10^−2^) was observed between these parameters and the fluctuation of effects. Compared to the independent effect, the synergetic effect resulted in a 12.5% reduction in average expression values, decreasing from 0.497 (±0.004) to 0.435 (±0.111). Among the proteins, 34 (30.1%) exhibited increased expression, while 79 (69.9%) showed decreased expression, a statistically significant change with a *P*-value of 1.42 × 10^−8^ (Fig. 8b and Supplementary Table S4). Notably, transitioning from the independent effect to the synergistic effect led to a 27.4-fold increase in the standard deviation of average expression values. Additionally, the differences between the two effects showed a strong correlation (~0.9, *P*-value = 1.55 × 10^−23^) with the node degree of proteins in the PPI network (Supplementary Table S5), underscoring the enhanced differentiation among proteins achieved by incorporating PPIs.

**Figure 9:**
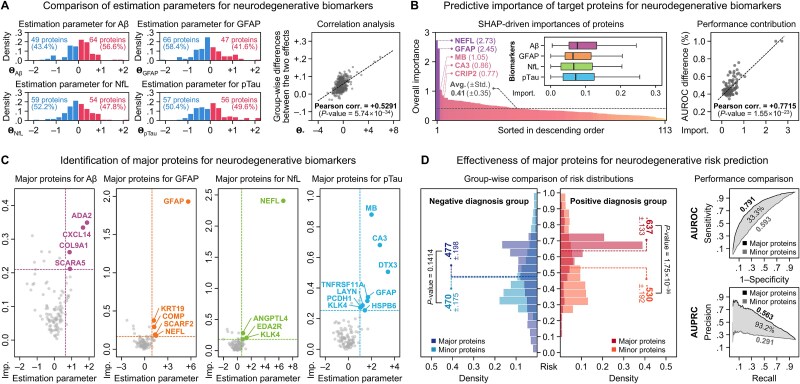
Predictive importance of the target proteins. (a) Compares the estimation parameters for target proteins, including the correlation between group-wise differences between the two effects and the estimation parameters. (b) Presents the SHAP-driven predictive importance of target proteins, along with the correlation between performance contribution and protein importance. (c) Identifies major proteins for neurodegenerative biomarkers and (d) evaluates their effectiveness in neurodegenerative risk prediction.

Furthermore, a comparison of the statistical significance between the independent and synergetic effects across diagnostic groups for neurodegenerative biomarkers revealed substantial improvement. The synergetic effect achieved an average *P*-value of 6.87, representing a 39.4% enhancement compared to the independent effect, which had an average *P*-value of 4.93 (Fig. 8c and Supplementary Table S6). Additionally, the propagation parameters were positively correlated with the –log_10_ *P*-values of target proteins in the synergetic effect (Pearson correlation = 0.3606, *P*-value = 8.71 × 10^−5^). These results demonstrate that the synergetic effect derived from PPIxGPN offers a significantly enhanced capacity to distinguish variations in protein expression and their associations with diagnostic groups, compared to the independent effect. The improved discrimination and increased statistical significance highlight the critical role of incorporating PPI networks in predicting neurodegenerative risks and capturing the intricate interactions among proteins.

#### Predictive importance of the target proteins

We further assessed the predictive importance of the target proteins for predicting neurodegenerative risks. First, a comparison of the estimation parameters for each biomarker revealed that, except for Aβ, more proteins exhibited negative values for the remaining three biomarkers (Fig. 9a and Supplementary Table S7). Overall, 48.9% of the proteins contributed to an increase in risks through positive estimation parameter values. Of the target proteins, 13 (11.5%) proteins showed positive estimation parameter values across all biomarkers, with NEFL and GFAP standing out with the most prominent sum of the parameters. Moreover, the estimation parameters showed a positive correlation (Pearson correlation = 0.5291, *P*-value = 5.74 × 10^−34^) with the diagnostic group-wise differences between the independent and synergetic effects. Next, the importances of target proteins for neurodegenerative risk prediction were evaluated using SHapley Additive exPlanations (SHAP) [57]. The average of importances was 0.41, with 41 proteins (36.3%) exceeding this threshold (Fig. 9b and Supplementary Table S8), and NEFL and GFAP scored the highest importances, at 2.73 and 2.45, respectively, followed by MB (1.05), CA3 (0.86), and CRIP2 (0.77). A significant positive correlation (Pearson correlation = 0.7313, *P*-value = 3.69 × 10^−20^) was observed between the protein importances and their individual contributions to prediction performance, measured by the change in AUROC when each protein was excluded from the synergetic effect.

**Figure 10:**
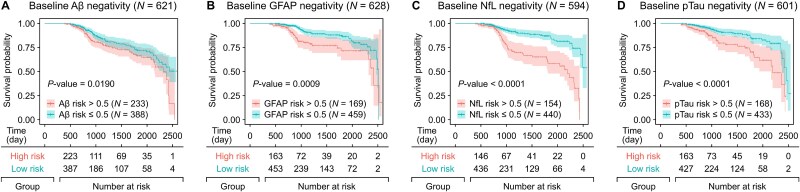
Survival analysis for longitudinal changes in neurodegenerative biomarkers. Kaplan–Meier survival curves (a–d) illustrate the progression from baseline negativity to follow-up positivity for high-risk and low-risk groups based on the predicted risk scores for Aβ, GFAP, NfL, and pTau, respectively. Participants with predicted risk scores exceeding 0.5 for each biomarker were classified as the high-risk group.

Additionally, the target proteins with significant impacts on increasing risks for neurodegenerative biomarkers were identified by combining estimation parameters and importance scores. For each biomarker, proteins within the top 10% for both metrics were selected as major proteins, resulting in a total of 19 major proteins: 4 for Aβ, 5 for GFAP, 4 for NfL, and 9 for pTau (Fig. 9c and Supplementary Table S9). Among these, three proteins—GFAP, NEFL, and KLK4—were selected as major proteins for two biomarkers each, where GFAP protein was selected for both GFAP and pTau, NEFL protein for both GFAP and NfL, and KLK4 protein for both NfL and pTau. Then, the effectiveness of these major proteins was validated by analyzing diagnostic group-wise differences in risk score distributions. As shown in Fig. 9d, the positive diagnosis group had an average risk score of 0.637 from major proteins that was 20.1% higher than that from minor proteins, while the negative diagnosis group showed a 1.6% increase. This resulted in a difference in average risk scores between diagnostic groups for major proteins (0.160), which was 164.6% greater than the difference observed for minor proteins (0.060). Furthermore, the risk scores derived from major proteins achieved an AUROC of 0.791 and an AUPRC of 0.562, representing improvements of 33.3% and 93.2%, respectively, compared to the risk scores derived from minor proteins. As a result, this analysis enabled the identification of the predictive contributions of proteins. In particular, by integrating estimation parameters and importance scores, 19 major proteins were identified as key contributors to risk prediction for neurodegenerative biomarkers, demonstrating stronger associations with diagnostic group discrimination and enhancing the predicted risk scores.

### Survival analysis

At last, we performed a survival analysis to investigate longitudinal changes associated with neurodegenerative biomarkers based on the predicted risk scores. Of the 758 participants with follow-up data, the baseline negative diagnosis groups comprised 621, 628, 594, and 601 participants for Aβ, GFAP, NfL, and pTau, respectively. Participants with predicted risk scores above 0.5 for each biomarker were classified as the high-risk group, including 233 participants for Aβ, 169 for GFAP, 154 for NfL, and 168 for pTau. As shown in Fig. 10, the results of this analysis revealed significant differences in survival probabilities between high-risk and low-risk groups, indicating that higher predicted risk scores are associated with markedly lower survival probabilities across all biomarkers. The hazard ratios for the biomarkers were derived as follows: Aβ at 1.507 (95% CI: 1.066–2.132), GFAP at 2.138 (95% CI: 1.351–3.381), NfL at 3.5 18 (95% CI: 2.296–5.390), and pTau at 2.443 (95% CI: 1.554–3.840), with NfL demonstrating the strongest association with survival outcomes. These findings suggest that the risks predicted by PPIxGPN are strongly linked to the progression of neurodegeneration, underscoring the capacity of PPIxGPN to enhance early detection of neurodegenerative diseases by effectively capturing dynamic changes in biomarker profiles over time.

## Conclusion

In this study, we introduce PPIxGPN, an explainable ML model that integrates PPI networks into plasma proteomic profiling of neurodegenerative biomarkers, offering two key advantages. First, unlike conventional ML approaches that assess proteins independently, PPIxGPN captures synergetic effects by integrating the independent effects of target proteins with the PPI network. Through globality-based feature aggregation, PPIxGPN constructs a comprehensive representation of the PPI network—particularly valuable for modeling blood-based biomarkers such as Aβ_42/40_, GFAP, NfL, and pTau_181_—since it identifies both individual expression levels and network-level interdependencies tied to disease risk. Experimental results on the UKB dataset confirm PPIxGPN’s superior performance over comparison methods. Second, PPIxGPN adopts a shallow, two-layer architecture (a single propagational layer followed by a fully connected layer), ensuring high explainability by reducing parameter complexity, clarifying each parameter’s function, and making the overall process easier to trace. Such intuitive design is especially critical in medical contexts, where clear insight into the biological understanding of model outputs is essential. Furthermore, by identifying major proteins for the neurodegenerative biomarkers and analyzing longitudinal changes in their diagnosis, PPIxGPN not only highlights the biological relevance of these proteins through interpretable outcomes but also effectively captures temporal shifts in biomarker profiles, underscoring its clinical applicability.

Here are some remarks on the method we proposed. First, the findings in this study are limited to a single cohort with internal validation due to the scarcity of public datasets that include both neurodegenerative biomarkers and plasma proteins. Future studies should validate PPIxGPN using external, multi-cohort datasets across diverse ethnic backgrounds to improve its robustness and generalizability. Second, PPIxGPN can be enhanced by constructing a more refined PPI network, considering the imbalanced nature of PPI characteristics to achieve a more sophisticated representation of protein data [58, 59]. Third, PPIxGPN can be further enriched into a biologically informed model by incorporating molecular pathways and functional annotations, allowing for a deeper understanding of disease mechanisms beyond protein interactions alone. Fourth, extending PPIxGPN into a multimodal model by integrating genetic information, neuroimaging data, and clinical parameters could enhance its ability to predict disease progression, offering more clinically meaningful insights for precision medicine. Last, to bridge the gap between research and clinical practice, further efforts should focus on adapting PPIxGPN for real-world applications, including the development of user-friendly clinical tools that allow healthcare professionals to interpret its predictions effectively.

**Key Points**

- We introduce Protein–Protein Interaction-based eXplainable Graph Propagational Network (PPIxGPN), a novel machine learning (ML) model designed for plasma proteomic profiling of neurodegenerative biomarkers, predicting individual risks for β-amyloid, glial fibrillary acidic protein, neurofilament light, and phosphorylated tau.
- PPIxGPN integrates the protein–protein interaction (PPI) network with the independent effects of proteins, capturing synergetic effects through globality-based feature aggregation, which enhances the comprehensive representation of PPI networks.
- PPIxGPN ensures high explainability with a shallow two-layer architecture, providing interpretable outputs that improve understanding of biological significance and disease progression.
- Experimental validation confirms PPIxGPN’s superior performance in predicting neurodegenerative risks, demonstrating its ability to identify biologically relevant proteins, generate explainable outcomes, and capture longitudinal biomarker changes.
- This study presents PPIxGPN as a powerful, explainable, and biologically informative ML model, offering improved predictive accuracy and clinical interpretability, with strong potential for early diagnosis and precision medicine applications in neurodegenerative diseases.

## Supplementary Material

PPIxGPN_SupplementaryData_bbaf213

## Data Availability

This research has been conducted using the UK Biobank Resource under Application Number 79011. Requests to access the data should be made via application to UK Biobank (https://www.ukbiobank.ac.uk/). The source codes of this study are available at https://github.com/sunghongpark-ai/PPIxGPN.
