# Supplementary material for: PPIxGPN: plasma proteomic profiling of neurodegenerative biomarkers with protein–protein interaction-based eXplainable graph propagational network
Source: Brief Bioinform. 2025 May 29;26(3):bbaf213. doi: 10.1093/bib/bbaf213 (PMC12121361; doi:10.1093/bib/bbaf213)
Supplement: PPIxGPN_SupplementaryData_bbaf213 [file ppixgpn_supplementarydata_bbaf213.docx]

Supplementary Data

PPIxGPN: plasma proteomic profiling of neurodegenerative biomarkers with protein–protein interaction-based explainable graph propagational network

Sunghong Park^1^^,^^†^, Dong-gi Lee^2^^,†^, Juhyeon Kim^3,4,†^, Seung Ho Kim^1,5^, Hyeon Jin Hwang^1,5^,

Hyunjung Shin^3,6,*^, Hyun Goo Woo^1,5,7,*^

^1^Department of Physiology, Ajou University School of Medicine, Suwon, 16499, Republic of Korea

^2^Department of Biostatistics, Epidemiology & Informatics, Perelman School of Medicine, University of Pennsylvania, Philadelphia, PA 19104, USA

^3^Department of Industrial Engineering, Ajou University, Suwon, 16499, Republic of Korea

^4^Department of Data–Centric Problem Solving Research, Korea Institute of Science and Technology Information, Daejeon, 34141, Republic of Korea

^5^Department of Biomedical Science, Graduate School of Ajou University, Suwon, 16499, Republic of Korea

^6^Department of Artificial Intelligence, Ajou University, Suwon, 16499, Republic of Korea

^7^Ajou Translational Omics Center, Research Institute for Innovative Medicine, Ajou University Medical Center, Suwon, 16499, Republic of Korea

^†^ These authors contributed equally to this work.

**^*^ Corresponding authors:** Hyunjung Shin ([shin@ajou.ac.kr](mailto:shin@ajou.ac.kr)) and Hyun Goo Woo ([hg@ajou.ac.kr](mailto:hg@ajou.ac.kr))

**Contents**

**Supplementary Figure S1.** PPI network for target proteins

**Supplementary Table S1.** Differentially expressed proteins for neurodegenerative biomarkers

**Supplementary Table S2.** Performance comparison for predicting neurodegenerative risks

**Supplementary Table S3.** Propagation parameters of target proteins

**Supplementary Table S4.** Comparison of protein expressions for independent and synergetic effects

**Supplementary Table S5.** Effect differences of target proteins and node degrees on PPI network

**Supplementary Table S6.** Comparison of P-values for independent and synergetic effects

**Supplementary Table S7.** Estimation parameter of target proteins for neurodegenerative biomarkers

**Supplementary Table S8.** Comparison between overall importance and performance contribution

**Supplementary Table S9.** Predictive importances of target proteins for neurodegenerative biomarkers


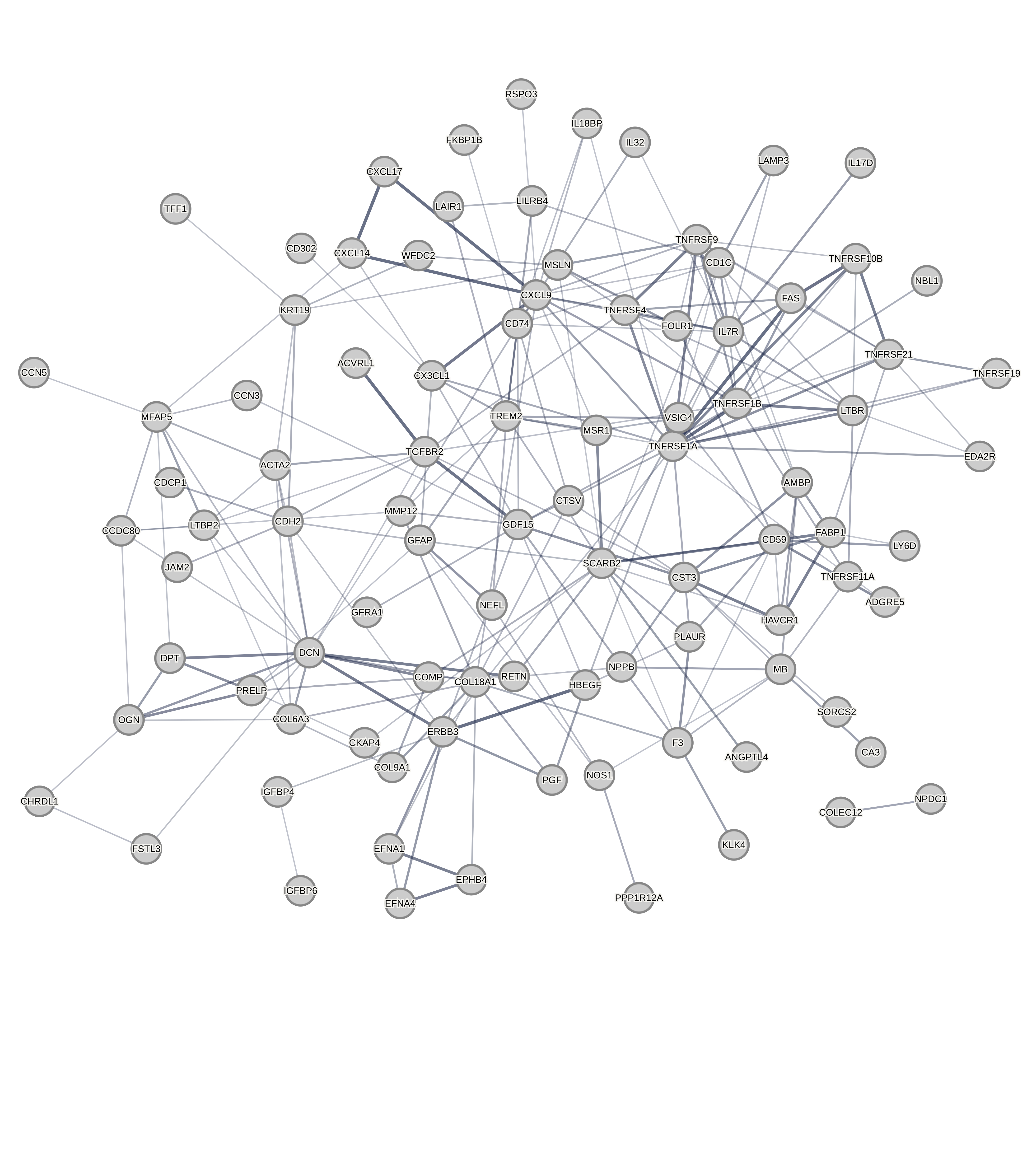


**Supplementary Figure S1.** PPI network for target proteins

**Supplementary Table S1.** Differentially expressed proteins for neurodegenerative biomarkers

| No. | Protein | log_2_Fold-Change | | | | –log_10_*P-*value | | | | No. | Protein | log_2_Fold-Change | | | | –log_10_*P-*value | | | |
| --- | --- | --- | --- | --- | --- | --- | --- | --- | --- | --- | --- | --- | --- | --- | --- | --- | --- | --- | --- |
|  |  | Aβ | GFAP | NfL | pTau | Aβ | GFAP | NfL | pTau |  |  | Aβ | GFAP | NfL | pTau | Aβ | GFAP | NfL | pTau |
| 1 | ACTA2 | 0.116 | 0.230 | 0.284 | 0.277 | 5.395 | 6.536 | 5.220 | 5.955 | 58 | IL17D | 0.085 | 0.247 | 0.294 | 0.127 | 3.908 | 6.640 | 6.025 | 4.273 |
| 2 | ACVRL1 | 0.084 | 0.168 | 0.232 | 0.179 | 3.655 | 1.924 | 1.463 | 2.172 | 59 | IL18BP | 0.093 | 0.150 | 0.201 | 0.207 | 3.265 | 1.850 | 1.834 | 2.235 |
| 3 | ADA2 | 0.138 | 0.146 | 0.131 | 0.063 | 3.387 | 1.925 | 2.813 | 1.938 | 60 | IL32 | 0.076 | 0.161 | 0.090 | 0.105 | 1.791 | 3.136 | 1.480 | 1.971 |
| 4 | ADGRE5 | 0.058 | 0.140 | 0.144 | 0.122 | 1.311 | 2.024 | 1.810 | 1.312 | 61 | IL7R | –0.151 | –0.106 | –0.067 | 0.019 | 2.375 | 2.568 | 3.181 | 1.449 |
| 5 | ADGRG1 | 0.105 | 0.086 | 0.137 | 0.104 | 2.096 | 1.692 | 3.046 | 3.874 | 62 | JAM2 | 0.030 | 0.212 | 0.233 | 0.338 | 3.420 | 3.013 | 2.528 | 3.451 |
| 6 | AMBP | 0.147 | 0.174 | 0.176 | 0.143 | 5.148 | 4.007 | 2.530 | 3.083 | 63 | KIR2DL3 | 0.050 | 0.079 | 0.176 | 0.124 | 1.613 | 1.313 | 1.306 | 2.639 |
| 7 | ANGPTL4 | 0.112 | 0.164 | 0.230 | 0.211 | 4.097 | 4.177 | 3.728 | 2.245 | 64 | KLK4 | 0.053 | 0.258 | 0.307 | 0.233 | 2.776 | 4.688 | 5.924 | 3.209 |
| 8 | BTN2A1 | 0.038 | 0.198 | 0.218 | 0.309 | 3.293 | 1.372 | 1.863 | 2.276 | 65 | KRT19 | 0.079 | 0.206 | 0.212 | 0.128 | 2.246 | 5.446 | 3.190 | 1.510 |
| 9 | CA3 | 0.057 | 0.104 | 0.161 | 0.413 | 3.352 | 2.991 | 2.227 | 2.569 | 66 | LAIR1 | 0.053 | 0.196 | 0.254 | 0.234 | 2.677 | 2.452 | 2.600 | 2.465 |
| 10 | CCDC80 | 0.104 | 0.287 | 0.297 | 0.216 | 4.485 | 5.910 | 5.652 | 5.693 | 67 | LAMP3 | 0.167 | 0.162 | 0.230 | 0.147 | 5.785 | 3.433 | 4.104 | 3.469 |
| 11 | CCN3 | 0.088 | 0.199 | 0.257 | 0.241 | 4.251 | 2.347 | 3.248 | 4.476 | 68 | LAYN | 0.015 | 0.300 | 0.345 | 0.319 | 3.207 | 3.361 | 2.432 | 3.397 |
| 12 | CCN5 | 0.074 | 0.128 | 0.262 | 0.240 | 2.826 | 3.747 | 3.384 | 4.569 | 69 | LGALS7 | 0.058 | 0.024 | 0.112 | 0.180 | 1.345 | 2.034 | 2.212 | 1.540 |
| 13 | CD1C | –0.152 | –0.112 | –0.137 | –0.011 | 2.669 | 1.562 | 2.566 | 1.946 | 70 | LILRB4 | 0.129 | 0.095 | 0.168 | 0.108 | 3.218 | 2.658 | 3.368 | 2.917 |
| 14 | CD302 | 0.143 | 0.159 | 0.225 | 0.208 | 4.839 | 3.905 | 5.120 | 4.098 | 71 | LRP11 | 0.035 | 0.129 | 0.187 | 0.226 | 1.805 | 1.408 | 1.541 | 2.658 |
| 15 | CD59 | 0.015 | 0.136 | 0.217 | 0.279 | 1.540 | 2.735 | 2.559 | 1.841 | 72 | LTBP2 | 0.000 | 0.335 | 0.327 | 0.216 | 1.317 | 7.402 | 4.479 | 2.995 |
| 16 | CD74 | 0.102 | 0.174 | 0.219 | 0.223 | 4.014 | 2.975 | 3.166 | 3.593 | 73 | LTBR | 0.044 | 0.176 | 0.184 | 0.298 | 2.484 | 2.067 | 2.404 | 1.857 |
| 17 | CDCP1 | 0.071 | 0.106 | 0.269 | 0.109 | 2.067 | 1.432 | 2.575 | 1.935 | 74 | LY6D | 0.036 | 0.086 | 0.201 | 0.264 | 1.443 | 3.209 | 3.908 | 2.512 |
| 18 | CDH2 | 0.097 | 0.125 | 0.137 | 0.151 | 2.557 | 1.476 | 1.605 | 2.581 | 75 | MB | 0.094 | 0.116 | 0.236 | 0.453 | 5.622 | 4.083 | 3.287 | 6.184 |
| 19 | CHRDL1 | 0.062 | 0.244 | 0.302 | 0.227 | 3.208 | 5.072 | 4.427 | 3.807 | 76 | MFAP5 | 0.051 | 0.026 | 0.129 | 0.215 | 1.758 | 1.389 | 1.536 | 1.786 |
| 20 | CKAP4 | 0.029 | 0.192 | 0.286 | 0.290 | 2.195 | 3.002 | 3.466 | 3.923 | 77 | MMP12 | 0.117 | 0.158 | 0.270 | 0.125 | 3.120 | 4.294 | 5.126 | 2.498 |
| 21 | COL18A1 | 0.049 | 0.142 | 0.171 | 0.123 | 1.369 | 2.543 | 2.586 | 1.811 | 78 | MSLN | 0.108 | 0.185 | 0.228 | 0.077 | 3.116 | 2.550 | 2.477 | 1.571 |
| 22 | COL6A3 | 0.029 | 0.152 | 0.234 | 0.195 | 1.654 | 1.857 | 1.457 | 2.035 | 79 | MSR1 | 0.110 | 0.106 | 0.198 | 0.174 | 3.654 | 3.328 | 3.336 | 1.958 |
| 23 | COL9A1 | 0.127 | 0.194 | 0.204 | 0.040 | 3.768 | 4.678 | 2.454 | 2.255 | 80 | NBL1 | 0.006 | 0.184 | 0.247 | 0.289 | 1.913 | 2.018 | 1.514 | 2.019 |
| 24 | COLEC12 | 0.069 | 0.256 | 0.277 | 0.238 | 4.117 | 3.013 | 2.421 | 2.292 | 81 | NEFL | 0.093 | 0.373 | 0.661 | 0.242 | 8.818 | 10.368 | 11.428 | 9.141 |
| 25 | COMP | 0.063 | 0.197 | 0.186 | 0.243 | 2.581 | 3.346 | 2.280 | 3.294 | 82 | NOS1 | 0.054 | 0.104 | 0.169 | 0.371 | 2.701 | 2.465 | 1.562 | 3.685 |
| 26 | CRIP2 | 0.015 | 0.029 | 0.141 | 0.258 | 1.442 | 2.104 | 1.417 | 1.465 | 83 | NPDC1 | –0.011 | 0.295 | 0.276 | 0.236 | 1.543 | 2.854 | 1.726 | 2.167 |
| 27 | CST3 | 0.068 | 0.146 | 0.215 | 0.223 | 3.032 | 1.322 | 1.315 | 2.955 | 84 | NPPB | 0.085 | 0.095 | 0.121 | 0.132 | 1.747 | 1.819 | 2.808 | 3.108 |
| 28 | CTSV | –0.094 | –0.137 | –0.257 | –0.068 | 2.692 | 2.672 | 2.488 | 1.970 | 85 | OGN | 0.061 | 0.230 | 0.245 | 0.115 | 2.484 | 4.084 | 2.597 | 2.462 |
| 29 | CX3CL1 | 0.063 | 0.138 | 0.129 | 0.211 | 2.090 | 2.928 | 1.746 | 1.406 | 86 | PCDH1 | 0.102 | 0.064 | 0.127 | 0.227 | 3.039 | 2.896 | 3.092 | 3.040 |
| 30 | CXCL14 | 0.184 | 0.136 | 0.199 | 0.011 | 5.044 | 6.726 | 6.427 | 3.402 | 87 | PGF | 0.108 | 0.121 | 0.241 | 0.214 | 3.930 | 3.283 | 4.099 | 5.024 |
| 31 | CXCL17 | 0.152 | 0.226 | 0.287 | 0.041 | 4.507 | 5.249 | 6.036 | 2.933 | 88 | PIK3IP1 | 0.054 | 0.254 | 0.334 | 0.307 | 4.618 | 3.364 | 3.878 | 3.180 |
| 32 | CXCL9 | 0.122 | 0.178 | 0.238 | 0.114 | 3.366 | 2.465 | 4.361 | 4.077 | 89 | PLAUR | 0.083 | 0.248 | 0.280 | 0.217 | 3.602 | 5.190 | 3.826 | 2.131 |
| 33 | DCN | 0.080 | 0.167 | 0.226 | 0.081 | 1.709 | 3.943 | 3.776 | 4.121 | 90 | PPP1R12A | –0.147 | 0.042 | –0.079 | 0.091 | 1.426 | 1.828 | 2.291 | 1.919 |
| 34 | DPT | 0.076 | 0.211 | 0.175 | 0.177 | 2.467 | 3.724 | 2.675 | 2.709 | 91 | PRELP | 0.093 | 0.246 | 0.228 | 0.221 | 4.348 | 5.939 | 3.189 | 3.740 |
| 35 | DSC2 | 0.065 | 0.192 | 0.270 | 0.249 | 3.750 | 2.301 | 1.627 | 2.255 | 92 | PSG1 | 0.049 | 0.169 | 0.194 | 0.156 | 1.803 | 3.169 | 1.307 | 2.761 |
| 36 | DTX3 | 0.010 | 0.214 | 0.213 | 0.419 | 4.097 | 3.192 | 2.161 | 1.819 | 93 | RETN | 0.060 | 0.067 | 0.149 | 0.207 | 1.705 | 1.400 | 1.720 | 2.274 |
| 37 | DUSP3 | –0.168 | 0.056 | –0.002 | 0.044 | 2.031 | 2.250 | 1.368 | 1.969 | 94 | RSPO3 | 0.008 | 0.310 | 0.285 | 0.252 | 2.123 | 4.403 | 4.580 | 1.587 |
| 38 | EDA2R | 0.069 | 0.308 | 0.447 | 0.308 | 5.590 | 8.246 | 7.194 | 6.100 | 95 | SCARA5 | 0.117 | 0.223 | 0.283 | 0.216 | 4.996 | 7.577 | 5.350 | 4.863 |
| 39 | EFNA1 | 0.055 | 0.170 | 0.234 | 0.183 | 2.381 | 1.524 | 2.349 | 1.903 | 96 | SCARB2 | 0.079 | 0.224 | 0.288 | 0.217 | 4.117 | 3.272 | 2.457 | 3.696 |
| 40 | EFNA4 | 0.096 | 0.147 | 0.205 | 0.200 | 3.620 | 1.583 | 1.755 | 2.768 | 97 | SCARF2 | 0.071 | 0.339 | 0.335 | 0.227 | 4.219 | 5.712 | 4.738 | 4.177 |
| 41 | EPHB4 | 0.047 | 0.179 | 0.195 | 0.242 | 2.439 | 2.744 | 1.681 | 1.497 | 98 | SORCS2 | 0.021 | 0.260 | 0.271 | 0.282 | 2.440 | 2.925 | 1.771 | 4.040 |
| 42 | ERBB3 | –0.120 | –0.019 | –0.137 | –0.082 | 2.108 | 2.295 | 1.934 | 3.320 | 99 | TFF1 | 0.046 | 0.176 | 0.229 | 0.144 | 1.656 | 1.660 | 1.543 | 1.495 |
| 43 | F3 | 0.050 | 0.201 | 0.179 | 0.234 | 2.231 | 2.716 | 1.570 | 1.509 | 100 | TGFBR2 | 0.091 | 0.185 | 0.257 | 0.276 | 4.735 | 1.960 | 2.497 | 3.405 |
| 44 | FABP1 | 0.038 | 0.185 | 0.182 | 0.146 | 1.507 | 3.896 | 1.628 | 2.295 | 101 | TNFRSF10B | 0.112 | 0.189 | 0.250 | 0.149 | 5.095 | 5.056 | 3.828 | 3.765 |
| 45 | FAS | 0.050 | 0.126 | 0.183 | 0.188 | 2.327 | 1.780 | 2.096 | 2.569 | 102 | TNFRSF11A | 0.061 | 0.119 | 0.213 | 0.335 | 3.491 | 1.878 | 2.510 | 2.297 |
| 46 | FKBP1B | –0.146 | 0.027 | –0.062 | 0.124 | 1.346 | 1.806 | 1.523 | 1.821 | 103 | TNFRSF19 | 0.019 | 0.213 | 0.277 | 0.356 | 3.091 | 2.928 | 2.213 | 2.737 |
| 47 | FOLR1 | 0.018 | 0.270 | 0.255 | 0.235 | 1.958 | 2.868 | 2.535 | 1.959 | 104 | TNFRSF1A | 0.089 | 0.174 | 0.255 | 0.292 | 5.140 | 2.446 | 2.826 | 3.109 |
| 48 | FSTL3 | 0.060 | 0.198 | 0.222 | 0.269 | 2.996 | 2.276 | 2.624 | 2.705 | 105 | TNFRSF1B | 0.074 | 0.172 | 0.212 | 0.185 | 3.454 | 2.308 | 1.811 | 1.655 |
| 49 | FUT3 | 0.111 | 0.055 | 0.174 | 0.037 | 1.586 | 3.764 | 4.020 | 1.922 | 106 | TNFRSF21 | 0.043 | 0.215 | 0.206 | 0.232 | 2.564 | 1.711 | 1.351 | 2.127 |
| 50 | GDF15 | 0.112 | 0.316 | 0.379 | 0.257 | 5.292 | 8.963 | 8.215 | 5.082 | 107 | TNFRSF4 | 0.133 | 0.144 | 0.182 | 0.144 | 4.770 | 3.148 | 2.347 | 2.186 |
| 51 | GFAP | 0.065 | 0.557 | 0.254 | 0.262 | 4.532 | 6.570 | 3.597 | 4.921 | 108 | TNFRSF9 | 0.125 | 0.140 | 0.189 | 0.144 | 4.382 | 2.664 | 2.829 | 2.342 |
| 52 | GFRA1 | 0.091 | 0.209 | 0.236 | 0.139 | 3.512 | 2.118 | 1.901 | 2.327 | 109 | TREM2 | 0.103 | 0.197 | 0.286 | 0.141 | 3.589 | 3.373 | 3.044 | 2.774 |
| 53 | HAVCR1 | 0.151 | 0.201 | 0.235 | 0.087 | 5.255 | 2.318 | 3.198 | 1.916 | 110 | TSPAN1 | 0.071 | 0.108 | 0.176 | 0.126 | 2.414 | 3.420 | 3.915 | 2.912 |
| 54 | HBEGF | –0.137 | –0.007 | –0.092 | 0.093 | 1.306 | 2.228 | 1.428 | 1.867 | 111 | VSIG4 | 0.064 | 0.145 | 0.250 | 0.243 | 3.281 | 1.965 | 2.121 | 2.766 |
| 55 | HSPB6 | 0.108 | 0.162 | 0.294 | 0.446 | 7.710 | 5.268 | 5.799 | 5.502 | 112 | WFDC2 | 0.140 | 0.322 | 0.394 | 0.294 | 8.398 | 7.226 | 8.003 | 6.907 |
| 56 | IGFBP4 | 0.060 | 0.225 | 0.310 | 0.249 | 4.082 | 3.256 | 2.867 | 3.276 | 113 | WNT9A | 0.071 | 0.264 | 0.303 | 0.213 | 3.171 | 7.303 | 4.388 | 2.203 |
| 57 | IGFBP6 | 0.035 | 0.112 | 0.250 | 0.273 | 2.579 | 1.540 | 1.916 | 3.320 |  |  |  |  |  |  |  |  |  |  |

**Supplementary Table S2.** Performance comparison for predicting neurodegenerative risks

| (a) Performance comparison for identifying Aβ | | | | | |
| --- | --- | --- | --- | --- | --- |
| Method | AUROC | AUPRC | Accuracy | F1-score | Average |
| Baseline | 0.539 | 0.226 | 0.524 | 0.319 | 0.402 |
| GCN | 0.552 | 0.238 | 0.530 | 0.330 | 0.413 |
| SGC | 0.556 | 0.241 | 0.530 | 0.336 | 0.416 |
| EGC | 0.573 | 0.252 | 0.534 | 0.341 | 0.425 |
| LGC | 0.576 | 0.255 | 0.536 | 0.342 | 0.427 |
| MixHop | 0.580 | 0.248 | 0.539 | 0.353 | 0.430 |
| UGCN | 0.584 | 0.254 | 0.542 | 0.353 | 0.433 |
| MOGCN | 0.587 | 0.257 | 0.552 | 0.362 | 0.439 |
| PPIxGPN | 0.620 | 0.301 | 0.572 | 0.362 | 0.464 |
| (b) Performance comparison for identifying GFAP | | | | | |
| Method | AUROC | AUPRC | Accuracy | F1-score | Average |
| Baseline | 0.650 | 0.277 | 0.568 | 0.378 | 0.468 |
| GCN | 0.699 | 0.383 | 0.597 | 0.407 | 0.521 |
| SGC | 0.720 | 0.385 | 0.627 | 0.433 | 0.541 |
| EGC | 0.753 | 0.425 | 0.655 | 0.469 | 0.575 |
| LGC | 0.760 | 0.437 | 0.649 | 0.459 | 0.576 |
| MixHop | 0.751 | 0.444 | 0.618 | 0.440 | 0.563 |
| UGCN | 0.752 | 0.443 | 0.620 | 0.438 | 0.563 |
| MOGCN | 0.760 | 0.457 | 0.642 | 0.453 | 0.578 |
| PPIxGPN | 0.798 | 0.503 | 0.689 | 0.496 | 0.622 |
| (c) Performance comparison for identifying NfL | | | | | |
| Method | AUROC | AUPRC | Accuracy | F1-score | Average |
| Baseline | 0.741 | 0.524 | 0.626 | 0.495 | 0.596 |
| GCN | 0.748 | 0.533 | 0.636 | 0.510 | 0.607 |
| SGC | 0.736 | 0.510 | 0.648 | 0.519 | 0.603 |
| EGC | 0.773 | 0.559 | 0.661 | 0.533 | 0.631 |
| LGC | 0.780 | 0.569 | 0.669 | 0.541 | 0.640 |
| MixHop | 0.796 | 0.610 | 0.673 | 0.550 | 0.657 |
| UGCN | 0.795 | 0.609 | 0.667 | 0.541 | 0.653 |
| MOGCN | 0.805 | 0.621 | 0.687 | 0.559 | 0.668 |
| PPIxGPN | 0.812 | 0.627 | 0.731 | 0.597 | 0.692 |
| (d) Performance comparison for identifying pTau | | | | | |
| Method | AUROC | AUPRC | Accuracy | F1-score | Average |
| Baseline | 0.669 | 0.417 | 0.619 | 0.455 | 0.540 |
| GCN | 0.704 | 0.424 | 0.610 | 0.466 | 0.551 |
| SGC | 0.757 | 0.513 | 0.648 | 0.493 | 0.603 |
| EGC | 0.766 | 0.527 | 0.651 | 0.495 | 0.610 |
| LGC | 0.769 | 0.534 | 0.652 | 0.494 | 0.613 |
| MixHop | 0.745 | 0.477 | 0.638 | 0.494 | 0.589 |
| UGCN | 0.747 | 0.477 | 0.641 | 0.498 | 0.591 |
| MOGCN | 0.751 | 0.485 | 0.652 | 0.504 | 0.598 |
| PPIxGPN | 0.762 | 0.517 | 0.684 | 0.514 | 0.619 |

**Supplementary Table S3.** Propagation parameters of target proteins

| No. | Protein | $\boldsymbol{\phi}$^*^ | No. | Protein | $\boldsymbol{\phi}$^*^ | No. | Protein | $\boldsymbol{\phi}$^*^ | No. | Protein | $\boldsymbol{\phi}$^*^ | No. | Protein | $\boldsymbol{\phi}$^*^ | No. | Protein | $\boldsymbol{\phi}$^*^ |
| --- | --- | --- | --- | --- | --- | --- | --- | --- | --- | --- | --- | --- | --- | --- | --- | --- | --- |
| 1 | ACTA2 | 1.772 | 20 | CKAP4 | 1.343 | 39 | EFNA1 | 0.598 | 58 | IL17D | 2.243 | 77 | MMP12 | 1.534 | 96 | SCARB2 | 1.106 |
| 2 | ACVRL1 | 0.901 | 21 | COL18A1 | 0.699 | 40 | EFNA4 | 0.574 | 59 | IL18BP | 0.785 | 78 | MSLN | 1.589 | 97 | SCARF2 | 1.324 |
| 3 | ADA2 | 1.644 | 22 | COL6A3 | 0.319 | 41 | EPHB4 | 0.521 | 60 | IL32 | 1.401 | 79 | MSR1 | 1.171 | 98 | SORCS2 | 1.575 |
| 4 | ADGRE5 | 0.827 | 23 | COL9A1 | 1.631 | 42 | ERBB3 | 0.902 | 61 | IL7R | 0.956 | 80 | NBL1 | 0.873 | 99 | TFF1 | 0.821 |
| 5 | ADGRG1 | 1.047 | 24 | COLEC12 | 0.416 | 43 | F3 | 1.610 | 62 | JAM2 | 1.005 | 81 | NEFL | 3.472 | 100 | TGFBR2 | 0.570 |
| 6 | AMBP | 1.196 | 25 | COMP | 1.663 | 44 | FABP1 | 1.005 | 63 | KIR2DL3 | 0.876 | 82 | NOS1 | 1.396 | 101 | TNFRSF10B | 1.272 |
| 7 | ANGPTL4 | 1.747 | 26 | CRIP2 | 2.004 | 45 | FAS | 1.001 | 64 | KLK4 | 2.192 | 83 | NPDC1 | 0.761 | 102 | TNFRSF11A | 1.820 |
| 8 | BTN2A1 | 1.549 | 27 | CST3 | 0.437 | 46 | FKBP1B | 1.104 | 65 | KRT19 | 1.725 | 84 | NPPB | 1.731 | 103 | TNFRSF19 | 1.699 |
| 9 | CA3 | 1.979 | 28 | CTSV | 1.313 | 47 | FOLR1 | 1.479 | 66 | LAIR1 | 0.864 | 85 | OGN | 0.823 | 104 | TNFRSF1A | 0.689 |
| 10 | CCDC80 | 1.871 | 29 | CX3CL1 | 0.892 | 48 | FSTL3 | 0.680 | 67 | LAMP3 | 1.440 | 86 | PCDH1 | 1.303 | 105 | TNFRSF1B | 0.588 |
| 11 | CCN3 | 0.975 | 30 | CXCL14 | 2.105 | 49 | FUT3 | 1.012 | 68 | LAYN | 1.472 | 87 | PGF | 0.917 | 106 | TNFRSF21 | 0.389 |
| 12 | CCN5 | 0.940 | 31 | CXCL17 | 1.674 | 50 | GDF15 | 2.150 | 69 | LGALS7 | 1.405 | 88 | PIK3IP1 | 1.379 | 107 | TNFRSF4 | 0.682 |
| 13 | CD1C | 0.922 | 32 | CXCL9 | 1.680 | 51 | GFAP | 2.677 | 70 | LILRB4 | 1.202 | 89 | PLAUR | 1.249 | 108 | TNFRSF9 | 0.720 |
| 14 | CD302 | 0.993 | 33 | DCN | 1.229 | 52 | GFRA1 | 0.982 | 71 | LRP11 | 0.970 | 90 | PPP1R12A | 1.310 | 109 | TREM2 | 1.769 |
| 15 | CD59 | 0.452 | 34 | DPT | 0.739 | 53 | HAVCR1 | 2.049 | 72 | LTBP2 | 2.372 | 91 | PRELP | 1.900 | 110 | TSPAN1 | 1.053 |
| 16 | CD74 | 0.489 | 35 | DSC2 | 0.650 | 54 | HBEGF | 0.937 | 73 | LTBR | 0.445 | 92 | PSG1 | 0.803 | 111 | VSIG4 | 0.632 |
| 17 | CDCP1 | 1.899 | 36 | DTX3 | 2.364 | 55 | HSPB6 | 1.283 | 74 | LY6D | 1.302 | 93 | RETN | 0.990 | 112 | WFDC2 | 1.859 |
| 18 | CDH2 | 1.006 | 37 | DUSP3 | 1.525 | 56 | IGFBP4 | 0.656 | 75 | MB | 1.622 | 94 | RSPO3 | 1.761 | 113 | WNT9A | 1.007 |
| 19 | CHRDL1 | 1.307 | 38 | EDA2R | 2.942 | 57 | IGFBP6 | 0.843 | 76 | MFAP5 | 0.344 | 95 | SCARA5 | 0.928 | ^*^Propagation parameter | | |

**Supplementary Table S4.** Comparison of protein expressions for independent and synergetic effects

| No. | Protein | Independent  effect | Synergetic  effect | No. | Protein | Independent  effect | Synergetic  effect | No. | Protein | Independent  effect | Synergetic  effect |
| --- | --- | --- | --- | --- | --- | --- | --- | --- | --- | --- | --- |
| 1 | ACTA2 | 0.494 | 0.497 | 39 | EFNA1 | 0.497 | 0.466 | 77 | MMP12 | 0.497 | 0.451 |
| 2 | ACVRL1 | 0.498 | 0.376 | 40 | EFNA4 | 0.500 | 0.447 | 78 | MSLN | 0.493 | 0.507 |
| 3 | ADA2 | 0.498 | 0.310 | 41 | EPHB4 | 0.495 | 0.463 | 79 | MSR1 | 0.502 | 0.450 |
| 4 | ADGRE5 | 0.496 | 0.387 | 42 | ERBB3 | 0.500 | 0.597 | 80 | NBL1 | 0.499 | 0.319 |
| 5 | ADGRG1 | 0.481 | 0.246 | 43 | F3 | 0.497 | 0.541 | 81 | NEFL | 0.496 | 0.471 |
| 6 | AMBP | 0.502 | 0.501 | 44 | FABP1 | 0.497 | 0.526 | 82 | NOS1 | 0.495 | 0.498 |
| 7 | ANGPTL4 | 0.499 | 0.390 | 45 | FAS | 0.489 | 0.501 | 83 | NPDC1 | 0.495 | 0.496 |
| 8 | BTN2A1 | 0.498 | 0.303 | 46 | FKBP1B | 0.497 | 0.334 | 84 | NPPB | 0.500 | 0.481 |
| 9 | CA3 | 0.496 | 0.411 | 47 | FOLR1 | 0.500 | 0.446 | 85 | OGN | 0.499 | 0.523 |
| 10 | CCDC80 | 0.495 | 0.453 | 48 | FSTL3 | 0.498 | 0.381 | 86 | PCDH1 | 0.495 | 0.280 |
| 11 | CCN3 | 0.496 | 0.363 | 49 | FUT3 | 0.501 | 0.252 | 87 | PGF | 0.498 | 0.432 |
| 12 | CCN5 | 0.500 | 0.331 | 50 | GDF15 | 0.494 | 0.566 | 88 | PIK3IP1 | 0.499 | 0.289 |
| 13 | CD1C | 0.502 | 0.521 | 51 | GFAP | 0.500 | 0.513 | 89 | PLAUR | 0.498 | 0.475 |
| 14 | CD302 | 0.501 | 0.319 | 52 | GFRA1 | 0.499 | 0.325 | 90 | PPP1R12A | 0.499 | 0.411 |
| 15 | CD59 | 0.496 | 0.616 | 53 | HAVCR1 | 0.498 | 0.491 | 91 | PRELP | 0.508 | 0.516 |
| 16 | CD74 | 0.499 | 0.524 | 54 | HBEGF | 0.496 | 0.466 | 92 | PSG1 | 0.510 | 0.227 |
| 17 | CDCP1 | 0.497 | 0.405 | 55 | HSPB6 | 0.495 | 0.278 | 93 | RETN | 0.497 | 0.430 |
| 18 | CDH2 | 0.496 | 0.579 | 56 | IGFBP4 | 0.497 | 0.416 | 94 | RSPO3 | 0.498 | 0.358 |
| 19 | CHRDL1 | 0.502 | 0.417 | 57 | IGFBP6 | 0.498 | 0.379 | 95 | SCARA5 | 0.501 | 0.241 |
| 20 | CKAP4 | 0.498 | 0.372 | 58 | IL17D | 0.492 | 0.401 | 96 | SCARB2 | 0.497 | 0.654 |
| 21 | COL18A1 | 0.501 | 0.591 | 59 | IL18BP | 0.495 | 0.353 | 97 | SCARF2 | 0.501 | 0.285 |
| 22 | COL6A3 | 0.496 | 0.452 | 60 | IL32 | 0.497 | 0.371 | 98 | SORCS2 | 0.500 | 0.368 |
| 23 | COL9A1 | 0.494 | 0.443 | 61 | IL7R | 0.505 | 0.634 | 99 | TFF1 | 0.489 | 0.330 |
| 24 | COLEC12 | 0.499 | 0.497 | 62 | JAM2 | 0.500 | 0.359 | 100 | TGFBR2 | 0.497 | 0.577 |
| 25 | COMP | 0.499 | 0.481 | 63 | KIR2DL3 | 0.497 | 0.232 | 101 | TNFRSF10B | 0.493 | 0.478 |
| 26 | CRIP2 | 0.497 | 0.331 | 64 | KLK4 | 0.495 | 0.414 | 102 | TNFRSF11A | 0.501 | 0.433 |
| 27 | CST3 | 0.497 | 0.638 | 65 | KRT19 | 0.494 | 0.508 | 103 | TNFRSF19 | 0.498 | 0.426 |
| 28 | CTSV | 0.495 | 0.413 | 66 | LAIR1 | 0.498 | 0.386 | 104 | TNFRSF1A | 0.498 | 0.726 |
| 29 | CX3CL1 | 0.498 | 0.500 | 67 | LAMP3 | 0.496 | 0.405 | 105 | TNFRSF1B | 0.495 | 0.612 |
| 30 | CXCL14 | 0.491 | 0.474 | 68 | LAYN | 0.498 | 0.297 | 106 | TNFRSF21 | 0.500 | 0.530 |
| 31 | CXCL17 | 0.497 | 0.445 | 69 | LGALS7 | 0.494 | 0.289 | 107 | TNFRSF4 | 0.499 | 0.511 |
| 32 | CXCL9 | 0.490 | 0.633 | 70 | LILRB4 | 0.499 | 0.438 | 108 | TNFRSF9 | 0.497 | 0.561 |
| 33 | DCN | 0.499 | 0.674 | 71 | LRP11 | 0.497 | 0.245 | 109 | TREM2 | 0.498 | 0.583 |
| 34 | DPT | 0.503 | 0.473 | 72 | LTBP2 | 0.495 | 0.479 | 110 | TSPAN1 | 0.479 | 0.245 |
| 35 | DSC2 | 0.500 | 0.197 | 73 | LTBR | 0.497 | 0.554 | 111 | VSIG4 | 0.501 | 0.420 |
| 36 | DTX3 | 0.492 | 0.345 | 74 | LY6D | 0.497 | 0.405 | 112 | WFDC2 | 0.495 | 0.431 |
| 37 | DUSP3 | 0.489 | 0.295 | 75 | MB | 0.493 | 0.546 | 113 | WNT9A | 0.497 | 0.249 |
| 38 | EDA2R | 0.499 | 0.437 | 76 | MFAP5 | 0.502 | 0.588 |  |  |  |  |

**Supplementary Table S5.** Effect differences of target proteins and node degrees on PPI network

| No. | Protein | Effect  difference | Node  degree | No. | Protein | Effect  difference | Node  degree | No. | Protein | Effect  difference | Node  degree |
| --- | --- | --- | --- | --- | --- | --- | --- | --- | --- | --- | --- |
| 1 | ACTA2 | +0.003 | 7 | 39 | EFNA1 | –0.030 | 4 | 77 | MMP12 | –0.046 | 6 |
| 2 | ACVRL1 | –0.122 | 1 | 40 | EFNA4 | –0.053 | 3 | 78 | MSLN | +0.014 | 6 |
| 3 | ADA2 | –0.188 | 0 | 41 | EPHB4 | –0.032 | 3 | 79 | MSR1 | –0.052 | 4 |
| 4 | ADGRE5 | –0.109 | 2 | 42 | ERBB3 | +0.098 | 9 | 80 | NBL1 | –0.180 | 1 |
| 5 | ADGRG1 | –0.235 | 0 | 43 | F3 | +0.043 | 7 | 81 | NEFL | –0.024 | 3 |
| 6 | AMBP | –0.001 | 6 | 44 | FABP1 | +0.028 | 6 | 82 | NOS1 | +0.003 | 4 |
| 7 | ANGPTL4 | –0.109 | 1 | 45 | FAS | +0.012 | 7 | 83 | NPDC1 | +0.001 | 1 |
| 8 | BTN2A1 | –0.195 | 0 | 46 | FKBP1B | –0.162 | 1 | 84 | NPPB | –0.019 | 5 |
| 9 | CA3 | –0.085 | 1 | 47 | FOLR1 | –0.054 | 3 | 85 | OGN | +0.024 | 6 |
| 10 | CCDC80 | –0.042 | 5 | 48 | FSTL3 | –0.117 | 2 | 86 | PCDH1 | –0.215 | 0 |
| 11 | CCN3 | –0.133 | 2 | 49 | FUT3 | –0.249 | 0 | 87 | PGF | –0.066 | 3 |
| 12 | CCN5 | –0.169 | 1 | 50 | GDF15 | +0.072 | 12 | 88 | PIK3IP1 | –0.210 | 0 |
| 13 | CD1C | +0.019 | 8 | 51 | GFAP | +0.012 | 7 | 89 | PLAUR | –0.023 | 5 |
| 14 | CD302 | –0.181 | 1 | 52 | GFRA1 | –0.175 | 1 | 90 | PPP1R12A | –0.088 | 1 |
| 15 | CD59 | +0.120 | 8 | 53 | HAVCR1 | –0.007 | 5 | 91 | PRELP | +0.009 | 6 |
| 16 | CD74 | +0.025 | 8 | 54 | HBEGF | –0.030 | 5 | 92 | PSG1 | –0.283 | 0 |
| 17 | CDCP1 | –0.092 | 1 | 55 | HSPB6 | –0.217 | 0 | 93 | RETN | –0.067 | 4 |
| 18 | CDH2 | +0.083 | 8 | 56 | IGFBP4 | –0.081 | 2 | 94 | RSPO3 | –0.140 | 1 |
| 19 | CHRDL1 | –0.085 | 2 | 57 | IGFBP6 | –0.120 | 1 | 95 | SCARA5 | –0.260 | 0 |
| 20 | CKAP4 | –0.126 | 2 | 58 | IL17D | –0.091 | 1 | 96 | SCARB2 | +0.156 | 15 |
| 21 | COL18A1 | +0.090 | 10 | 59 | IL18BP | –0.142 | 3 | 97 | SCARF2 | –0.215 | 0 |
| 22 | COL6A3 | –0.044 | 6 | 60 | IL32 | –0.126 | 2 | 98 | SORCS2 | –0.132 | 1 |
| 23 | COL9A1 | –0.051 | 3 | 61 | IL7R | +0.129 | 14 | 99 | TFF1 | –0.159 | 1 |
| 24 | COLEC12 | –0.002 | 1 | 62 | JAM2 | –0.140 | 1 | 100 | TGFBR2 | +0.079 | 9 |
| 25 | COMP | –0.017 | 5 | 63 | KIR2DL3 | –0.265 | 0 | 101 | TNFRSF10B | –0.015 | 6 |
| 26 | CRIP2 | –0.165 | 0 | 64 | KLK4 | –0.081 | 1 | 102 | TNFRSF11A | –0.068 | 3 |
| 27 | CST3 | +0.140 | 9 | 65 | KRT19 | +0.014 | 5 | 103 | TNFRSF19 | –0.073 | 3 |
| 28 | CTSV | –0.082 | 3 | 66 | LAIR1 | –0.112 | 2 | 104 | TNFRSF1A | +0.228 | 19 |
| 29 | CX3CL1 | +0.002 | 7 | 67 | LAMP3 | –0.091 | 2 | 105 | TNFRSF1B | +0.117 | 13 |
| 30 | CXCL14 | –0.017 | 4 | 68 | LAYN | –0.201 | 0 | 106 | TNFRSF21 | +0.030 | 8 |
| 31 | CXCL17 | –0.052 | 2 | 69 | LGALS7 | –0.205 | 0 | 107 | TNFRSF4 | +0.012 | 9 |
| 32 | CXCL9 | +0.142 | 16 | 70 | LILRB4 | –0.061 | 3 | 108 | TNFRSF9 | +0.064 | 11 |
| 33 | DCN | +0.175 | 16 | 71 | LRP11 | –0.252 | 0 | 109 | TREM2 | +0.085 | 11 |
| 34 | DPT | –0.030 | 4 | 72 | LTBP2 | –0.016 | 6 | 110 | TSPAN1 | –0.233 | 0 |
| 35 | DSC2 | –0.303 | 0 | 73 | LTBR | 0.057 | 9 | 111 | VSIG4 | –0.081 | 4 |
| 36 | DTX3 | –0.146 | 0 | 74 | LY6D | –0.091 | 2 | 112 | WFDC2 | –0.064 | 2 |
| 37 | DUSP3 | –0.194 | 0 | 75 | MB | +0.053 | 7 | 113 | WNT9A | –0.248 | 0 |
| 38 | EDA2R | –0.062 | 3 | 76 | MFAP5 | +0.086 | 8 |  |  |  |  |

**Supplementary Table S6.** Comparison of *P*-values for independent and synergetic effects

| No. | Protein | –log_10_*P*-value | | No. | Protein | –log_10_*P*-value | | No. | Protein | –log_10_*P*-value | |
| --- | --- | --- | --- | --- | --- | --- | --- | --- | --- | --- | --- |
|  |  | Independent  effect | Synergetic  effect |  |  | Independent  effect | Synergetic  effect |  |  | Independent  effect | Synergetic  effect |
| 1 | ACTA2 | 7.442 | 10.001 | 39 | EFNA1 | 3.836 | 5.425 | 77 | MMP12 | 4.190 | 6.599 |
| 2 | ACVRL1 | 3.852 | 6.140 | 40 | EFNA4 | 3.611 | 5.231 | 78 | MSLN | 3.315 | 6.390 |
| 3 | ADA2 | 2.106 | 2.106 | 41 | EPHB4 | 4.055 | 6.071 | 79 | MSR1 | 3.216 | 5.644 |
| 4 | ADGRE5 | 2.027 | 4.446 | 42 | ERBB3 | 1.384 | 1.735 | 80 | NBL1 | 5.630 | 6.724 |
| 5 | ADGRG1 | 2.132 | 2.132 | 43 | F3 | 4.099 | 8.410 | 81 | NEFL | 23.736 | 25.636 |
| 6 | AMBP | 3.353 | 6.116 | 44 | FABP1 | 2.837 | 6.728 | 82 | NOS1 | 6.108 | 9.872 |
| 7 | ANGPTL4 | 4.291 | 5.487 | 45 | FAS | 4.165 | 7.508 | 83 | NPDC1 | 6.721 | 7.868 |
| 8 | BTN2A1 | 5.730 | 5.730 | 46 | FKBP1B | 1.313 | 1.445 | 84 | NPPB | 1.869 | 4.882 |
| 9 | CA3 | 6.885 | 8.561 | 47 | FOLR1 | 5.902 | 7.591 | 85 | OGN | 4.059 | 8.207 |
| 10 | CCDC80 | 7.169 | 8.714 | 48 | FSTL3 | 5.035 | 7.251 | 86 | PCDH1 | 2.980 | 2.980 |
| 11 | CCN3 | 5.320 | 7.468 | 49 | FUT3 | 1.661 | 1.661 | 87 | PGF | 4.176 | 4.905 |
| 12 | CCN5 | 4.784 | 6.059 | 50 | GDF15 | 9.799 | 11.841 | 88 | PIK3IP1 | 8.465 | 8.465 |
| 13 | CD1C | 1.734 | 0.924 | 51 | GFAP | 13.769 | 17.984 | 89 | PLAUR | 5.926 | 8.309 |
| 14 | CD302 | 4.510 | 5.624 | 52 | GFRA1 | 3.940 | 6.074 | 90 | PPP1R12A | 1.274 | 2.099 |
| 15 | CD59 | 4.562 | 9.443 | 53 | HAVCR1 | 4.028 | 5.871 | 91 | PRELP | 6.608 | 9.349 |
| 16 | CD74 | 4.461 | 6.366 | 54 | HBEGF | 1.180 | 1.928 | 92 | PSG1 | 3.516 | 3.516 |
| 17 | CDCP1 | 3.351 | 4.182 | 55 | HSPB6 | 10.491 | 10.491 | 93 | RETN | 2.605 | 5.951 |
| 18 | CDH2 | 2.220 | 7.349 | 56 | IGFBP4 | 6.625 | 6.966 | 94 | RSPO3 | 7.125 | 7.606 |
| 19 | CHRDL1 | 6.685 | 7.602 | 57 | IGFBP6 | 4.847 | 6.076 | 95 | SCARA5 | 6.033 | 6.033 |
| 20 | CKAP4 | 6.365 | 7.998 | 58 | IL17D | 6.598 | 6.791 | 96 | SCARB2 | 5.768 | 10.100 |
| 21 | COL18A1 | 2.285 | 6.588 | 59 | IL18BP | 3.729 | 5.321 | 97 | SCARF2 | 8.479 | 8.479 |
| 22 | COL6A3 | 3.665 | 9.181 | 60 | IL32 | 1.828 | 2.491 | 98 | SORCS2 | 6.837 | 7.613 |
| 23 | COL9A1 | 3.125 | 4.800 | 61 | IL7R | 1.282 | 2.752 | 99 | TFF1 | 3.495 | 5.275 |
| 24 | COLEC12 | 6.216 | 8.131 | 62 | JAM2 | 6.743 | 8.271 | 100 | TGFBR2 | 5.724 | 10.781 |
| 25 | COMP | 4.268 | 6.437 | 63 | KIR2DL3 | 1.961 | 1.961 | 101 | TNFRSF10B | 5.708 | 8.025 |
| 26 | CRIP2 | 2.943 | 2.943 | 64 | KLK4 | 6.885 | 8.168 | 102 | TNFRSF11A | 5.646 | 7.365 |
| 27 | CST3 | 3.851 | 10.390 | 65 | KRT19 | 3.492 | 6.837 | 103 | TNFRSF19 | 7.651 | 8.740 |
| 28 | CTSV | 3.113 | 1.348 | 66 | LAIR1 | 4.953 | 6.345 | 104 | TNFRSF1A | 5.907 | 9.686 |
| 29 | CX3CL1 | 2.780 | 8.268 | 67 | LAMP3 | 4.011 | 3.922 | 105 | TNFRSF1B | 4.196 | 8.467 |
| 30 | CXCL14 | 3.194 | 5.199 | 68 | LAYN | 9.444 | 9.444 | 106 | TNFRSF21 | 4.507 | 10.387 |
| 31 | CXCL17 | 5.017 | 6.611 | 69 | LGALS7 | 1.858 | 1.858 | 107 | TNFRSF4 | 2.979 | 6.414 |
| 32 | CXCL9 | 3.823 | 7.027 | 70 | LILRB4 | 2.250 | 3.747 | 108 | TNFRSF9 | 2.986 | 6.814 |
| 33 | DCN | 3.003 | 7.783 | 71 | LRP11 | 3.290 | 3.290 | 109 | TREM2 | 4.782 | 8.581 |
| 34 | DPT | 3.550 | 7.434 | 72 | LTBP2 | 8.002 | 9.525 | 110 | TSPAN1 | 3.030 | 3.030 |
| 35 | DSC2 | 5.574 | 5.574 | 73 | LTBR | 4.776 | 9.401 | 111 | VSIG4 | 4.995 | 7.518 |
| 36 | DTX3 | 9.656 | 9.656 | 74 | LY6D | 3.842 | 5.462 | 112 | WFDC2 | 11.561 | 12.436 |
| 37 | DUSP3 | 1.150 | 1.150 | 75 | MB | 9.472 | 13.157 | 113 | WNT9A | 6.632 | 6.632 |
| 38 | EDA2R | 12.823 | 13.334 | 76 | MFAP5 | 2.311 | 9.653 |  |  |  |  |

**Supplementary Table S7.** Estimation parameter of target proteins for neurodegenerative biomarkers

| No. | Protein | Estimation parameter for biomarkers | | | | No. | Protein | Estimation parameter for biomarkers | | | |
| --- | --- | --- | --- | --- | --- | --- | --- | --- | --- | --- | --- |
|  |  | Aβ | GFAP | NfL | pTau |  |  | Aβ | GFAP | NfL | pTau |
| 1 | ACTA2 | +0.835 | –0.096 | +0.037 | +0.042 | 58 | IL17D | –0.010 | +0.601 | +0.976 | –0.035 |
| 2 | ACVRL1 | –0.153 | –0.276 | –0.522 | –0.887 | 59 | IL18BP | +0.064 | –0.238 | –0.152 | +0.408 |
| 3 | ADA2 | +1.888 | +0.118 | –0.700 | –0.785 | 60 | IL32 | +0.615 | –0.405 | –1.382 | –0.116 |
| 4 | ADGRE5 | –0.378 | +0.053 | –0.275 | –0.634 | 61 | IL7R | –1.147 | –1.316 | –0.627 | –0.689 |
| 5 | ADGRG1 | +0.246 | –0.503 | –0.689 | –0.655 | 62 | JAM2 | +0.158 | –0.161 | –0.700 | +0.991 |
| 6 | AMBP | +0.680 | +0.298 | –0.279 | –0.272 | 63 | KIR2DL3 | –0.013 | –0.703 | +0.299 | +0.295 |
| 7 | ANGPTL4 | +0.955 | +0.054 | +0.824 | +0.104 | 64 | KLK4 | +0.077 | +0.317 | +0.768 | +1.036 |
| 8 | BTN2A1 | –0.062 | +0.863 | +0.558 | +1.687 | 65 | KRT19 | +0.297 | +1.177 | +0.341 | –0.306 |
| 9 | CA3 | +0.064 | +0.171 | –0.054 | +2.732 | 66 | LAIR1 | –0.047 | +0.080 | +0.191 | –0.025 |
| 10 | CCDC80 | +0.434 | +0.565 | +0.339 | +0.092 | 67 | LAMP3 | +0.390 | –0.142 | –0.139 | –0.390 |
| 11 | CCN3 | +0.339 | –0.331 | –0.095 | –0.062 | 68 | LAYN | –1.123 | +0.437 | +0.758 | +1.233 |
| 12 | CCN5 | –0.476 | –0.997 | –0.345 | +0.013 | 69 | LGALS7 | –0.207 | –1.380 | –0.833 | –0.876 |
| 13 | CD1C | –1.088 | –1.484 | –1.063 | –0.504 | 70 | LILRB4 | +0.385 | –0.590 | –0.990 | –0.362 |
| 14 | CD302 | +0.704 | –0.151 | –0.299 | +0.025 | 71 | LRP11 | –0.295 | –0.700 | –0.318 | +0.562 |
| 15 | CD59 | –0.485 | –0.292 | –0.149 | –0.254 | 72 | LTBP2 | –1.271 | +0.763 | +0.418 | +0.418 |
| 16 | CD74 | –0.205 | –0.033 | –0.156 | +0.307 | 73 | LTBR | +0.006 | –0.029 | –0.513 | +0.132 |
| 17 | CDCP1 | –1.239 | –1.136 | +0.767 | +0.076 | 74 | LY6D | –0.305 | –1.433 | –0.051 | +0.445 |
| 18 | CDH2 | +0.449 | +0.056 | –0.278 | +0.193 | 75 | MB | +0.374 | –0.240 | +0.143 | +2.007 |
| 19 | CHRDL1 | +0.095 | –0.542 | +0.390 | +0.306 | 76 | MFAP5 | +0.097 | –0.802 | –0.495 | –0.276 |
| 20 | CKAP4 | –0.799 | –0.228 | +0.541 | +0.415 | 77 | MMP12 | +0.178 | –0.518 | +0.253 | –0.433 |
| 21 | COL18A1 | +0.209 | –0.520 | –0.230 | –0.540 | 78 | MSLN | +0.342 | –0.066 | –0.011 | –0.719 |
| 22 | COL6A3 | –0.274 | –0.395 | –0.011 | –0.586 | 79 | MSR1 | +0.308 | –0.335 | –0.119 | +0.457 |
| 23 | COL9A1 | +0.881 | +0.205 | +0.284 | –0.835 | 80 | NBL1 | –0.597 | –0.044 | +0.076 | +0.197 |
| 24 | COLEC12 | –0.216 | +1.019 | +0.689 | –0.290 | 81 | NEFL | +0.306 | +1.486 | +6.651 | +0.012 |
| 25 | COMP | +0.124 | +1.239 | +0.573 | +0.671 | 82 | NOS1 | +0.057 | –0.175 | –0.759 | +0.924 |
| 26 | CRIP2 | –1.185 | –2.304 | –1.184 | +0.215 | 83 | NPDC1 | –0.643 | +1.241 | +0.967 | –0.434 |
| 27 | CST3 | +0.139 | –0.162 | –0.259 | +0.027 | 84 | NPPB | +0.723 | –1.048 | –0.790 | +0.316 |
| 28 | CTSV | –0.444 | –0.927 | –1.997 | –0.862 | 85 | OGN | +0.217 | +0.157 | –0.190 | –0.632 |
| 29 | CX3CL1 | +0.770 | +0.011 | –0.409 | +0.169 | 86 | PCDH1 | +0.815 | –0.211 | –0.630 | +1.111 |
| 30 | CXCL14 | +1.654 | +0.738 | +0.807 | –1.293 | 87 | PGF | +0.095 | –0.404 | +0.062 | –0.535 |
| 31 | CXCL17 | +0.448 | –0.374 | –0.278 | –1.167 | 88 | PIK3IP1 | –0.454 | +0.838 | +1.225 | +0.549 |
| 32 | CXCL9 | +0.164 | +0.022 | +0.377 | +0.131 | 89 | PLAUR | +0.038 | +0.634 | +0.405 | +0.034 |
| 33 | DCN | +0.052 | –0.616 | –0.426 | –1.059 | 90 | PPP1R12A | –0.213 | –0.535 | –1.461 | –0.614 |
| 34 | DPT | +0.284 | +0.388 | –0.554 | –0.011 | 91 | PRELP | +0.920 | +1.046 | +0.148 | +0.850 |
| 35 | DSC2 | –0.007 | –0.352 | +0.134 | –0.059 | 92 | PSG1 | +0.210 | +0.036 | +0.264 | +0.596 |
| 36 | DTX3 | –0.103 | +0.726 | +0.163 | +3.436 | 93 | RETN | +0.380 | –0.683 | +0.093 | +0.252 |
| 37 | DUSP3 | –1.576 | +0.350 | +0.111 | –1.180 | 94 | RSPO3 | –1.357 | +1.047 | –0.079 | +0.374 |
| 38 | EDA2R | –1.130 | –0.112 | +1.326 | +0.224 | 95 | SCARA5 | +0.902 | –0.111 | –0.062 | +0.031 |
| 39 | EFNA1 | +0.147 | –0.080 | +0.139 | –0.407 | 96 | SCARB2 | –0.295 | +0.438 | +0.056 | +0.050 |
| 40 | EFNA4 | +0.398 | –0.047 | –0.194 | –0.204 | 97 | SCARF2 | +0.115 | +1.362 | +0.598 | –0.627 |
| 41 | EPHB4 | +0.234 | –0.086 | –0.198 | –0.093 | 98 | SORCS2 | –0.424 | +0.324 | +0.100 | +0.899 |
| 42 | ERBB3 | –0.743 | –0.548 | –0.670 | –0.745 | 99 | TFF1 | +0.094 | –0.232 | +0.116 | –0.442 |
| 43 | F3 | +0.275 | –0.057 | –0.237 | +0.204 | 100 | TGFBR2 | +0.341 | –0.169 | –0.010 | –0.123 |
| 44 | FABP1 | –0.421 | +0.424 | +0.121 | –0.214 | 101 | TNFRSF10B | +0.330 | +0.481 | –0.148 | –0.882 |
| 45 | FAS | –0.014 | –0.280 | –0.204 | –0.400 | 102 | TNFRSF11A | +0.370 | –0.627 | +0.323 | +1.642 |
| 46 | FKBP1B | –0.422 | –0.304 | –0.267 | +0.282 | 103 | TNFRSF19 | –0.442 | +0.050 | +0.182 | +0.883 |
| 47 | FOLR1 | –0.048 | –0.121 | –0.198 | +0.321 | 104 | TNFRSF1A | +0.141 | –0.032 | –0.252 | –0.206 |
| 48 | FSTL3 | –0.138 | –0.262 | –0.304 | +0.177 | 105 | TNFRSF1B | –0.187 | +0.252 | –0.171 | –0.376 |
| 49 | FUT3 | –0.011 | –0.817 | +0.082 | –0.807 | 106 | TNFRSF21 | –0.217 | +0.097 | –0.149 | –0.095 |
| 50 | GDF15 | –0.300 | +0.960 | +0.591 | +0.288 | 107 | TNFRSF4 | +0.699 | –0.175 | –0.278 | –0.562 |
| 51 | GFAP | +0.528 | +5.662 | +0.457 | +1.700 | 108 | TNFRSF9 | +0.391 | –0.039 | –0.403 | –0.467 |
| 52 | GFRA1 | +0.323 | +0.261 | +0.298 | –0.588 | 109 | TREM2 | +0.276 | +0.154 | +0.619 | +0.202 |
| 53 | HAVCR1 | +0.735 | +0.174 | +0.549 | –0.289 | 110 | TSPAN1 | +0.442 | –0.074 | +0.196 | –0.994 |
| 54 | HBEGF | –0.318 | –0.386 | –0.469 | –0.023 | 111 | VSIG4 | –0.279 | –0.287 | –0.135 | –0.085 |
| 55 | HSPB6 | –0.332 | +0.042 | +0.657 | +1.427 | 112 | WFDC2 | +0.653 | +1.235 | +0.753 | +1.155 |
| 56 | IGFBP4 | –0.372 | –0.040 | +0.027 | –0.370 | 113 | WNT9A | +0.084 | +0.989 | +0.312 | +0.263 |
| 57 | IGFBP6 | –0.701 | –0.531 | +0.209 | –0.566 |  |  |  |  |  |  |

**Supplementary Table S8.** Comparison between overall importance and performance contribution

| No. | Protein | Overall  importance | Performance  contribution | No. | Protein | Overall  importance | Performance  contribution | No. | Protein | Overall  importance | Performance  contribution |
| --- | --- | --- | --- | --- | --- | --- | --- | --- | --- | --- | --- |
| 1 | ACTA2 | 0.297 | 0.447 | 39 | EFNA1 | 0.285 | 0.404 | 77 | MMP12 | 0.198 | 0.390 |
| 2 | ACVRL1 | 0.371 | 0.449 | 40 | EFNA4 | 0.292 | 0.412 | 78 | MSLN | 0.259 | 0.376 |
| 3 | ADA2 | 0.671 | 0.525 | 41 | EPHB4 | 0.079 | 0.411 | 79 | MSR1 | 0.407 | 0.461 |
| 4 | ADGRE5 | 0.333 | 0.414 | 42 | ERBB3 | 0.435 | 0.434 | 80 | NBL1 | 0.339 | 0.461 |
| 5 | ADGRG1 | 0.236 | 0.392 | 43 | F3 | 0.178 | 0.420 | 81 | NEFL | 2.728 | 0.999 |
| 6 | AMBP | 0.436 | 0.429 | 44 | FABP1 | 0.232 | 0.454 | 82 | NOS1 | 0.703 | 0.475 |
| 7 | ANGPTL4 | 0.580 | 0.464 | 45 | FAS | 0.187 | 0.404 | 83 | NPDC1 | 0.473 | 0.538 |
| 8 | BTN2A1 | 0.286 | 0.614 | 46 | FKBP1B | 0.507 | 0.393 | 84 | NPPB | 0.374 | 0.491 |
| 9 | CA3 | 0.859 | 0.615 | 47 | FOLR1 | 0.242 | 0.456 | 85 | OGN | 0.367 | 0.379 |
| 10 | CCDC80 | 0.283 | 0.409 | 48 | FSTL3 | 0.206 | 0.448 | 86 | PCDH1 | 0.643 | 0.472 |
| 11 | CCN3 | 0.217 | 0.426 | 49 | FUT3 | 0.405 | 0.400 | 87 | PGF | 0.186 | 0.406 |
| 12 | CCN5 | 0.493 | 0.496 | 50 | GDF15 | 0.089 | 0.535 | 88 | PIK3IP1 | 0.205 | 0.497 |
| 13 | CD1C | 0.682 | 0.564 | 51 | GFAP | 2.450 | 0.998 | 89 | PLAUR | 0.144 | 0.436 |
| 14 | CD302 | 0.169 | 0.397 | 52 | GFRA1 | 0.212 | 0.390 | 90 | PPP1R12A | 0.610 | 0.468 |
| 15 | CD59 | 0.284 | 0.480 | 53 | HAVCR1 | 0.327 | 0.414 | 91 | PRELP | 0.478 | 0.546 |
| 16 | CD74 | 0.188 | 0.453 | 54 | HBEGF | 0.385 | 0.402 | 92 | PSG1 | 0.469 | 0.439 |
| 17 | CDCP1 | 0.544 | 0.636 | 55 | HSPB6 | 0.485 | 0.462 | 93 | RETN | 0.507 | 0.440 |
| 18 | CDH2 | 0.156 | 0.434 | 56 | IGFBP4 | 0.177 | 0.425 | 94 | RSPO3 | 0.367 | 0.653 |
| 19 | CHRDL1 | 0.234 | 0.474 | 57 | IGFBP6 | 0.186 | 0.505 | 95 | SCARA5 | 0.308 | 0.397 |
| 20 | CKAP4 | 0.350 | 0.554 | 58 | IL17D | 0.632 | 0.462 | 96 | SCARB2 | 0.130 | 0.456 |
| 21 | COL18A1 | 0.174 | 0.408 | 59 | IL18BP | 0.113 | 0.447 | 97 | SCARF2 | 0.340 | 0.395 |
| 22 | COL6A3 | 0.172 | 0.419 | 60 | IL32 | 0.587 | 0.448 | 98 | SORCS2 | 0.528 | 0.515 |
| 23 | COL9A1 | 0.653 | 0.414 | 61 | IL7R | 0.606 | 0.594 | 99 | TFF1 | 0.435 | 0.405 |
| 24 | COLEC12 | 0.348 | 0.420 | 62 | JAM2 | 0.333 | 0.525 | 100 | TGFBR2 | 0.161 | 0.410 |
| 25 | COMP | 0.516 | 0.562 | 63 | KIR2DL3 | 0.282 | 0.447 | 101 | TNFRSF10B | 0.308 | 0.403 |
| 26 | CRIP2 | 0.772 | 0.636 | 64 | KLK4 | 0.632 | 0.528 | 102 | TNFRSF11A | 0.696 | 0.675 |
| 27 | CST3 | 0.127 | 0.409 | 65 | KRT19 | 0.671 | 0.496 | 103 | TNFRSF19 | 0.194 | 0.521 |
| 28 | CTSV | 0.399 | 0.391 | 66 | LAIR1 | 0.258 | 0.417 | 104 | TNFRSF1A | 0.151 | 0.395 |
| 29 | CX3CL1 | 0.341 | 0.464 | 67 | LAMP3 | 0.332 | 0.401 | 105 | TNFRSF1B | 0.069 | 0.400 |
| 30 | CXCL14 | 0.703 | 0.626 | 68 | LAYN | 0.534 | 0.605 | 106 | TNFRSF21 | 0.168 | 0.422 |
| 31 | CXCL17 | 0.340 | 0.345 | 69 | LGALS7 | 0.360 | 0.464 | 107 | TNFRSF4 | 0.319 | 0.412 |
| 32 | CXCL9 | 0.283 | 0.442 | 70 | LILRB4 | 0.236 | 0.434 | 108 | TNFRSF9 | 0.195 | 0.385 |
| 33 | DCN | 0.312 | 0.410 | 71 | LRP11 | 0.304 | 0.465 | 109 | TREM2 | 0.338 | 0.452 |
| 34 | DPT | 0.273 | 0.406 | 72 | LTBP2 | 0.584 | 0.724 | 110 | TSPAN1 | 0.510 | 0.393 |
| 35 | DSC2 | 0.452 | 0.425 | 73 | LTBR | 0.049 | 0.442 | 111 | VSIG4 | 0.344 | 0.429 |
| 36 | DTX3 | 0.771 | 0.756 | 74 | LY6D | 0.414 | 0.554 | 112 | WFDC2 | 0.389 | 0.640 |
| 37 | DUSP3 | 0.679 | 0.404 | 75 | MB | 1.046 | 0.597 | 113 | WNT9A | 0.424 | 0.439 |
| 38 | EDA2R | 0.556 | 0.657 | 76 | MFAP5 | 0.086 | 0.445 |  |  |  |  |

**Supplementary Table S9.** Predictive importances of target proteins for neurodegenerative biomarkers

| No. | Protein | Predictive importance for biomarkers | | | | No. | Protein | Predictive importance for biomarkers | | | |
| --- | --- | --- | --- | --- | --- | --- | --- | --- | --- | --- | --- |
|  |  | Aβ | GFAP | NfL | pTau |  |  | Aβ | GFAP | NfL | pTau |
| 1 | ACTA2 | 0.131 | 0.089 | 0.017 | 0.059 | 58 | IL17D | 0.090 | 0.290 | 0.172 | 0.079 |
| 2 | ACVRL1 | 0.068 | 0.055 | 0.077 | 0.170 | 59 | IL18BP | 0.010 | 0.028 | 0.018 | 0.058 |
| 3 | ADA2 | 0.349 | 0.025 | 0.126 | 0.171 | 60 | IL32 | 0.113 | 0.082 | 0.305 | 0.087 |
| 4 | ADGRE5 | 0.094 | 0.023 | 0.079 | 0.136 | 61 | IL7R | 0.231 | 0.204 | 0.097 | 0.074 |
| 5 | ADGRG1 | 0.034 | 0.047 | 0.097 | 0.058 | 62 | JAM2 | 0.030 | 0.051 | 0.044 | 0.208 |
| 6 | AMBP | 0.186 | 0.138 | 0.040 | 0.071 | 63 | KIR2DL3 | 0.052 | 0.084 | 0.086 | 0.060 |
| 7 | ANGPTL4 | 0.149 | 0.091 | 0.280 | 0.061 | 64 | KLK4 | 0.063 | 0.115 | 0.181 | 0.272 |
| 8 | BTN2A1 | 0.052 | 0.047 | 0.055 | 0.131 | 65 | KRT19 | 0.099 | 0.374 | 0.131 | 0.068 |
| 9 | CA3 | 0.030 | 0.049 | 0.099 | 0.681 | 66 | LAIR1 | 0.028 | 0.065 | 0.135 | 0.029 |
| 10 | CCDC80 | 0.146 | 0.018 | 0.061 | 0.058 | 67 | LAMP3 | 0.133 | 0.093 | 0.029 | 0.076 |
| 11 | CCN3 | 0.091 | 0.048 | 0.054 | 0.024 | 68 | LAYN | 0.089 | 0.056 | 0.103 | 0.286 |
| 12 | CCN5 | 0.093 | 0.077 | 0.144 | 0.179 | 69 | LGALS7 | 0.075 | 0.105 | 0.063 | 0.116 |
| 13 | CD1C | 0.210 | 0.303 | 0.092 | 0.078 | 70 | LILRB4 | 0.061 | 0.062 | 0.095 | 0.017 |
| 14 | CD302 | 0.046 | 0.036 | 0.042 | 0.044 | 71 | LRP11 | 0.053 | 0.126 | 0.044 | 0.082 |
| 15 | CD59 | 0.169 | 0.032 | 0.037 | 0.047 | 72 | LTBP2 | 0.310 | 0.130 | 0.104 | 0.040 |
| 16 | CD74 | 0.029 | 0.031 | 0.090 | 0.039 | 73 | LTBR | 0.013 | 0.000 | 0.009 | 0.027 |
| 17 | CDCP1 | 0.165 | 0.093 | 0.132 | 0.153 | 74 | LY6D | 0.059 | 0.265 | 0.022 | 0.068 |
| 18 | CDH2 | 0.080 | 0.014 | 0.054 | 0.008 | 75 | MB | 0.088 | 0.046 | 0.032 | 0.880 |
| 19 | CHRDL1 | 0.030 | 0.151 | 0.022 | 0.031 | 76 | MFAP5 | 0.015 | 0.042 | 0.029 | 0.000 |
| 20 | CKAP4 | 0.071 | 0.125 | 0.109 | 0.045 | 77 | MMP12 | 0.038 | 0.049 | 0.040 | 0.071 |
| 21 | COL18A1 | 0.016 | 0.083 | 0.013 | 0.062 | 78 | MSLN | 0.052 | 0.097 | 0.061 | 0.048 |
| 22 | COL6A3 | 0.040 | 0.043 | 0.013 | 0.076 | 79 | MSR1 | 0.244 | 0.043 | 0.075 | 0.045 |
| 23 | COL9A1 | 0.261 | 0.168 | 0.125 | 0.100 | 80 | NBL1 | 0.235 | 0.042 | 0.032 | 0.030 |
| 24 | COLEC12 | 0.057 | 0.141 | 0.081 | 0.069 | 81 | NEFL | 0.070 | 0.176 | 2.405 | 0.076 |
| 25 | COMP | 0.043 | 0.291 | 0.068 | 0.114 | 82 | NOS1 | 0.059 | 0.037 | 0.289 | 0.318 |
| 26 | CRIP2 | 0.168 | 0.378 | 0.118 | 0.109 | 83 | NPDC1 | 0.135 | 0.112 | 0.102 | 0.124 |
| 27 | CST3 | 0.068 | 0.023 | 0.022 | 0.014 | 84 | NPPB | 0.116 | 0.028 | 0.122 | 0.108 |
| 28 | CTSV | 0.102 | 0.062 | 0.180 | 0.054 | 85 | OGN | 0.092 | 0.069 | 0.105 | 0.101 |
| 29 | CX3CL1 | 0.131 | 0.050 | 0.030 | 0.130 | 86 | PCDH1 | 0.127 | 0.088 | 0.150 | 0.278 |
| 30 | CXCL14 | 0.334 | 0.171 | 0.091 | 0.107 | 87 | PGF | 0.062 | 0.026 | 0.039 | 0.059 |
| 31 | CXCL17 | 0.094 | 0.035 | 0.146 | 0.065 | 88 | PIK3IP1 | 0.077 | 0.082 | 0.025 | 0.021 |
| 32 | CXCL9 | 0.062 | 0.087 | 0.103 | 0.031 | 89 | PLAUR | 0.042 | 0.051 | 0.016 | 0.035 |
| 33 | DCN | 0.089 | 0.027 | 0.016 | 0.180 | 90 | PPP1R12A | 0.043 | 0.103 | 0.342 | 0.121 |
| 34 | DPT | 0.124 | 0.082 | 0.038 | 0.029 | 91 | PRELP | 0.187 | 0.160 | 0.022 | 0.110 |
| 35 | DSC2 | 0.144 | 0.129 | 0.084 | 0.094 | 92 | PSG1 | 0.077 | 0.074 | 0.120 | 0.198 |
| 36 | DTX3 | 0.058 | 0.072 | 0.135 | 0.506 | 93 | RETN | 0.084 | 0.169 | 0.134 | 0.120 |
| 37 | DUSP3 | 0.167 | 0.161 | 0.180 | 0.172 | 94 | RSPO3 | 0.149 | 0.111 | 0.027 | 0.081 |
| 38 | EDA2R | 0.196 | 0.116 | 0.203 | 0.040 | 95 | SCARA5 | 0.211 | 0.036 | 0.046 | 0.013 |
| 39 | EFNA1 | 0.048 | 0.028 | 0.021 | 0.188 | 96 | SCARB2 | 0.058 | 0.062 | 0.000 | 0.009 |
| 40 | EFNA4 | 0.032 | 0.042 | 0.093 | 0.125 | 97 | SCARF2 | 0.059 | 0.190 | 0.074 | 0.017 |
| 41 | EPHB4 | 0.025 | 0.032 | 0.022 | 0.000 | 98 | SORCS2 | 0.172 | 0.065 | 0.145 | 0.146 |
| 42 | ERBB3 | 0.263 | 0.100 | 0.050 | 0.022 | 99 | TFF1 | 0.097 | 0.117 | 0.189 | 0.031 |
| 43 | F3 | 0.072 | 0.051 | 0.025 | 0.030 | 100 | TGFBR2 | 0.110 | 0.027 | 0.006 | 0.018 |
| 44 | FABP1 | 0.068 | 0.052 | 0.097 | 0.015 | 101 | TNFRSF10B | 0.057 | 0.138 | 0.028 | 0.086 |
| 45 | FAS | 0.092 | 0.032 | 0.030 | 0.033 | 102 | TNFRSF11A | 0.070 | 0.159 | 0.152 | 0.315 |
| 46 | FKBP1B | 0.100 | 0.047 | 0.061 | 0.300 | 103 | TNFRSF19 | 0.047 | 0.048 | 0.057 | 0.042 |
| 47 | FOLR1 | 0.055 | 0.066 | 0.063 | 0.059 | 104 | TNFRSF1A | 0.004 | 0.065 | 0.068 | 0.015 |
| 48 | FSTL3 | 0.028 | 0.115 | 0.021 | 0.042 | 105 | TNFRSF1B | 0.022 | 0.010 | 0.025 | 0.011 |
| 49 | FUT3 | 0.064 | 0.084 | 0.153 | 0.104 | 106 | TNFRSF21 | 0.032 | 0.061 | 0.029 | 0.045 |
| 50 | GDF15 | 0.031 | 0.023 | 0.015 | 0.020 | 107 | TNFRSF4 | 0.169 | 0.000 | 0.058 | 0.093 |
| 51 | GFAP | 0.141 | 1.931 | 0.037 | 0.340 | 108 | TNFRSF9 | 0.101 | 0.053 | 0.007 | 0.034 |
| 52 | GFRA1 | 0.060 | 0.035 | 0.014 | 0.103 | 109 | TREM2 | 0.077 | 0.030 | 0.199 | 0.032 |
| 53 | HAVCR1 | 0.123 | 0.035 | 0.033 | 0.136 | 110 | TSPAN1 | 0.057 | 0.058 | 0.282 | 0.114 |
| 54 | HBEGF | 0.153 | 0.093 | 0.097 | 0.043 | 111 | VSIG4 | 0.262 | 0.047 | 0.020 | 0.016 |
| 55 | HSPB6 | 0.076 | 0.014 | 0.141 | 0.254 | 112 | WFDC2 | 0.076 | 0.115 | 0.068 | 0.129 |
| 56 | IGFBP4 | 0.062 | 0.021 | 0.021 | 0.074 | 113 | WNT9A | 0.038 | 0.152 | 0.089 | 0.145 |
| 57 | IGFBP6 | 0.062 | 0.048 | 0.026 | 0.050 |  |  |  |  |  |  |
